# Through-Thickness Microhardness and Microstructure in Laser-Processed Polypropylene vs. Poly(ethylene terephthalate): A Comparative Study

**DOI:** 10.3390/ma19214438

**Published:** 2026-10-22

**Authors:** André Guimarães, André Costa Vieira, Vera L. D. Costa, Joana Costa Vieira, Tânia Ferreira

**Affiliations:** 1 CISE—Electomechatronic Systems Research Centre, University of Beira Interior, Rua Marquês de Ávila e Bolama, 6201-001 Covilhã, Portugal; andre.m.guimaraes@ubi.pt; 2 CISeD—Research Centre for Digital Services, Polytechnic Institute of Viseu, Av. Cor. José Maria Vale de Andrade, 3504-510 Viseu, Portugal; 3 CMAST—Centre for Mechanical and Aerospace Science and Technologies, University of Beira Interior, Rua Marquês de Ávila e Bolama, 6201-001 Covilhã, Portugal; 4 Fibentech—Fiber Materials and Environmental Technologies Research Unit, University da Beira Interior, R. Marquês D’Ávila e Bolama, 6201-001 Covilhã, Portugal; vera.costa@ubi.pt (V.L.D.C.); joana.costa.vieira@ubi.pt (J.C.V.); 5 Department of Mechanical Engineering and Industrial Management, Polytechnic Institute of Viseu, Av. Cor. José Maria Vale de Andrade, 3504-510 Viseu, Portugal; taniaferreira@estgv.ipv.pt

**Keywords:** polypropylene, polyethylene terephthalate, laser processing, crystallinity

## Abstract

This study compares the response of two widely used semicrystalline polymers, polypropylene (PP) and poly(ethylene terephthalate) (PET), to laser processing under identical conditions, focusing on through-thickness microhardness and microstructural evolution. The novelty lies in the direct comparison of both materials by integrating microhardness measurements with X-ray diffraction (XRD), Fourier transform infrared spectroscopy (FTIR), and differential scanning calorimetry (DSC) to relate mechanical behavior to structural and chemical changes. PP exhibited greater sensitivity to laser processing, particularly at low scanning speeds and higher power, with pronounced changes in the through-thickness hardness profile and early signs of thermo-oxidative effects. In contrast, PET showed more uniform hardness profiles and lower sensitivity to laser power, despite localized surface damage. Structural analyses indicated laser-induced changes in PET crystallinity consistent with melting and subsequent structural reorganization, while FTIR showed no clear evidence of significant chemical degradation. Overall, the results demonstrate distinct responses of PP and PET to laser-induced thermal input and highlight the importance of material-specific optimization of laser processing conditions.

## 1. Introduction

The joining of polymers presents an increasing challenge in sectors such as automotive, aerospace, and biomedical engineering [1,2], which demand lightweight, complex components manufactured through fast and reproducible methods. To ensure the effectiveness of such processes in industrial applications, it is essential to employ high-precision and highly repeatable technologies, such as laser welding [3].

Laser welding stands out as an emerging technology due to its high precision, resulting not only from the quality of the laser beam but also from its compatibility with robotic systems [4,5,6]. It is a non-contact process in which heating occurs by radiation, concentrating energy in specific regions without causing mechanical wear to the parts [7]. Additionally, the thermal impact can be locally controlled, limiting thermal gradients and overheating of adjacent regions [8]. Several authors have highlighted laser welding as an effective method for joining thermoplastic polymers owing to its precision, cleanliness, and versatility [4,9,10,11], with potential for integration into automated and repetitive manufacturing processes.

Despite these advantages, the localized thermal input associated with laser processing can modify polymer crystallinity [12], affecting thermal and mechanical properties [13]. Melt-pool morphology also depends on the material response to the laser beam and influences microstructural distribution and joint integrity [14]. Although the heat-affected zone (HAZ) is generally limited in well-controlled processes, it remains an important factor to monitor [7], together with thermal degradation, which may compromise polymer-chain stability [9]. These phenomena can directly affect the through-thickness hardness profile [15], an aspect that remains insufficiently investigated in semicrystalline polymers [16,17].

### 1.1. Laser Processing of Semicrystalline Polymers

Laser welding of polymers encompasses different techniques, including transmission welding (LTW), direct welding, and oscillatory welding, offering advantages over conventional methods such as ultrasonic welding, thermal bonding, and adhesives [9,10]. The most influential processing parameters include laser power, scanning speed, focal diameter, and exposure time [18]. The interaction between laser energy and scanning speed is particularly important because it directly influences the thermal input and the resulting surface morphology [19]. Process efficiency also depends on material-related characteristics, including absorptance and the presence of additives [10], the optical transmission index that determines the amount of energy reaching the interface [20], polymer viscosity in the molten state [9], and thermal residence time [21]. Understanding the resulting temperature fields, residual stresses, and melt flow is therefore important for controlling the process and limiting defects such as porosity and thermal degradation [17].

The influence of processing parameters on melt-pool geometry and the HAZ is well established [22]. Increasing laser power generally increases the amount of thermal energy delivered to the material, whereas higher scanning speeds reduce interaction time and tend to limit penetration and HAZ extension [9,11]. These variations affect cooling behavior, residual stresses, and consequently local hardness [23,24]. For PP, Guimarães et al. [11] showed that higher scanning speeds (≥30 mm/s) produce more uniform hardness profiles and reduce surface degradation, highlighting the importance of balancing absorbed energy and thermal exposure time. PET processing is likewise sensitive to laser intensity at the surface [25], while thermal and visual monitoring can contribute to improved process control and repeatability [10].

In semicrystalline polymers such as PP and PET, low thermal conductivity and the sensitivity of crystallinity to elevated temperatures make the response particularly dependent on the power–speed combination. Variations in these parameters can modify melt depth and HAZ extension, directly affecting the through-thickness microhardness profile [11,26]. Studies on PP have shown that controlling power, scanning speed, and beam diameter can produce stable melt regions, narrower HAZs, and more uniform hardness profiles [27]. Similar control is important for PET to obtain homogeneous microstructures while limiting thermal degradation [17,25].

The microstructure of semicrystalline polymers, defined by the degree of crystallinity, crystal size and orientation, and the proportion of amorphous regions, plays an important role in determining their mechanical and thermal properties. During laser processing, localized heating can induce structural reorganization and, under sufficiently severe thermal conditions, degradation [28]. Recrystallization may modify local hardness and modulus, whereas polymer-chain scission and oxidation can reduce molecular weight and mechanical strength [9,11,29].

Characterization techniques provide complementary information for assessing these laser-induced modifications. X-ray diffraction (XRD) can identify changes in crystalline structure resulting from thermal exposure [30,31], while Fourier transform infrared spectroscopy (FTIR) provides information on chemical changes associated with degradation and differential scanning calorimetry (DSC) evaluates thermal transitions and structural modifications. Combining these techniques with microhardness measurements therefore allows the relationship between laser-induced thermal input, structural evolution, and local mechanical response to be assessed [9,32,33].

### 1.2. PP and PET: Comparative Background and Research Gap

Among semicrystalline thermoplastics, polypropylene (PP) and poly(ethylene terephthalate) (PET) are particularly relevant because of their thermal, chemical, mechanical, and processing characteristics [34,35]. Their increasing industrial use is associated with their relatively low cost, mechanical performance, and thermal resistance [36,37]. However, their molecular structures and thermal properties differ considerably, resulting in distinct responses to thermal processing [9,29,34,38,39,40]. Thermal response and heat dissipation in thermoplastics depend strongly on molecular structure and crystallinity [16,41], making a direct comparison of PP and PET under identical laser conditions particularly relevant.

Compared with PP, PET exhibits higher density, stiffness, tensile strength, and higher glass-transition and melting temperatures [42]. Differences in thermal expansion, thermal conductivity, and melting behavior can influence dimensional stability, heat distribution, softening, and deformation during processing [42,43]. PP, with its lower melting temperature, is particularly sensitive to thermal input, while its crystallinity requires appropriate temperature control to limit degradation [44]. PET, despite its greater resistance to elevated temperatures, also operates within a relatively narrow processing window in which excessive thermal input can promote defects [45,46].

Previous research has examined PET in metal–polymer hybrid joints [47] and LTW thermal simulations [17], as well as its optical behavior [9,10,48], melt-pool formation, and joint strength [7]. Nevertheless, systematic experimental analyses combining through-thickness microhardness, crystallinity, HAZ morphology, and chemical changes remain limited. PP has been more extensively investigated in laser processing, including the previous work of Guimarães et al. [11], but direct comparisons between PP and PET processed under equivalent conditions remain scarce.

The two polymers also exhibit different thermal degradation mechanisms. At elevated temperatures, PET can undergo cleavage of C–C and C–O bonds, producing volatile compounds such as CO, CO_2_, and acetaldehyde that may contribute to bubble formation and surface damage [12,49,50]. Bubble nucleation may be further intensified by high energy input [51]. In contrast, PP degrades mainly through chain-scission mechanisms, generating aliphatic hydrocarbons with comparatively limited gas evolution [50,52,53,54,55]. These differences further support the expectation of distinct responses when the two materials are subjected to identical laser processing conditions.

Despite the growing interest in laser processing of polymers [56], direct experimental comparisons between PP and PET under identical processing conditions remain scarce, particularly those combining through-thickness microhardness with structural, chemical, and thermal characterization. Previous work by Guimarães et al. [11] provided detailed information on the response of PP; however, a direct comparison with PET processed under the same conditions is still lacking. This limits the understanding of how the distinct thermal and structural characteristics of these polymers influence their response to equivalent laser-induced thermal inputs.

The present study addresses this gap by directly comparing PP and PET under identical laser processing conditions, extending the previous investigation of PP by Guimarães et al. [11] to PET processed at the same power levels (200 and 300 W) and scanning speeds (5–50 mm/s). The central objective is to correlate the through-thickness microhardness response with the structural and chemical changes induced by laser processing. Microhardness measurements and optical microscopy are therefore combined with XRD, FTIR, and DSC analyses to evaluate HAZ development, structural evolution, chemical changes, and thermal behavior. This integrated approach enables a systematic comparison of the response of both polymers to equivalent processing conditions and provides a basis for understanding the relationship between laser parameters, microstructural evolution, and local mechanical behavior.

## 2. Materials and Methods

The laser treatment of PP and PET samples was performed according to the methodology established by Guimarães et al. [11]. A robot-assisted system guided multiple passes of a continuous-wave laser beam over the sample surface, ensuring precise control of beam position and overlap. The process was conducted at two power levels (200 W and 300 W) and six scanning speeds (5, 10, 20, 30, 40, and 50 mm/s) to evaluate the influence of laser parameters on the through-thickness hardness and microstructural evolution of both polymers.

**Figure 1:**
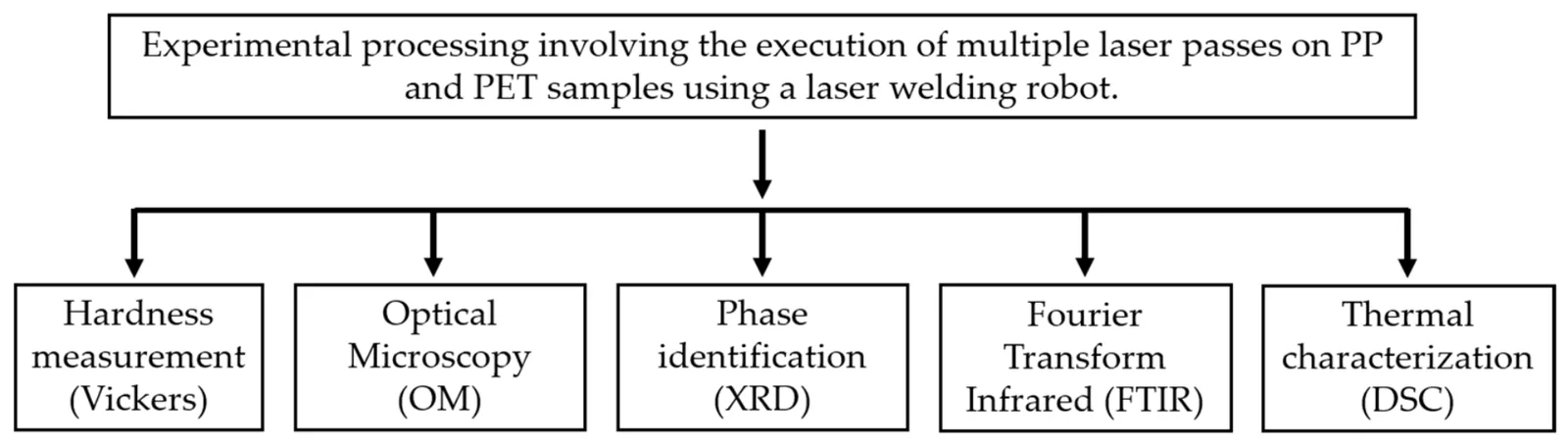
Flowchart of the experimental procedure.

The flowchart presented in Figure 1 summarizes the experimental procedure followed for hardness measurements, optical microscopy, phase identification (XRD) analyses and FTIR. It outlines the sequential steps performed after laser processing, including sample preparation, surface cleaning, and sectioning, followed by microstructural observation on optical microscope to measure the thickness of HAZ, XRD, FTIR spectra, and DSC. This workflow ensured consistent sample handling and reliable correlation between microstructural, thermal, and structural results.

### 2.1. Materials and Specimens

Commercial grades of PP and PET generally contain antioxidant packages designed to limit thermo-oxidative degradation during processing and service life. These additives may influence the response of polymers to laser-induced thermal exposure by reducing free-radical formation and delaying oxidation reactions. Both PP and PET used, are semicrystalline polymers composed of amorphous and crystalline regions, whose degree of crystallinity depends strongly on their thermal history. Commercial engineering-grade plates of PP and PET were supplied by Röchling Industrial (Haren, Germany) as Polystone^®^ P Homopolymer Grey and SUSTADUR^®^ PET, respectively. Both materials were used as received, without any additional thermal or surface treatment. The physical, mechanical and thermal properties summarized in Table 1 were obtained from the manufacturer’s technical datasheets. The manufacturer does not disclose the molecular weight or synthesis conditions of these commercial grades. Likewise, detailed information on the additive packages used in these commercial formulations, including the presence and concentration of antioxidants or other stabilizers, was not provided by the manufacturer and was not independently quantified in this study.

### 2.2. Laser and Processing Parameters

The laser processing of the PET samples followed the same experimental procedure previously established for PP by Guimarães et al. [11]. The same processing parameters were intentionally applied to both polymers to enable a direct comparison of their responses under identical laser energy inputs. Therefore, the selected conditions were not intended to represent an optimized processing window for either material, but rather a common experimental window for comparative analysis.

**Figure 2:**
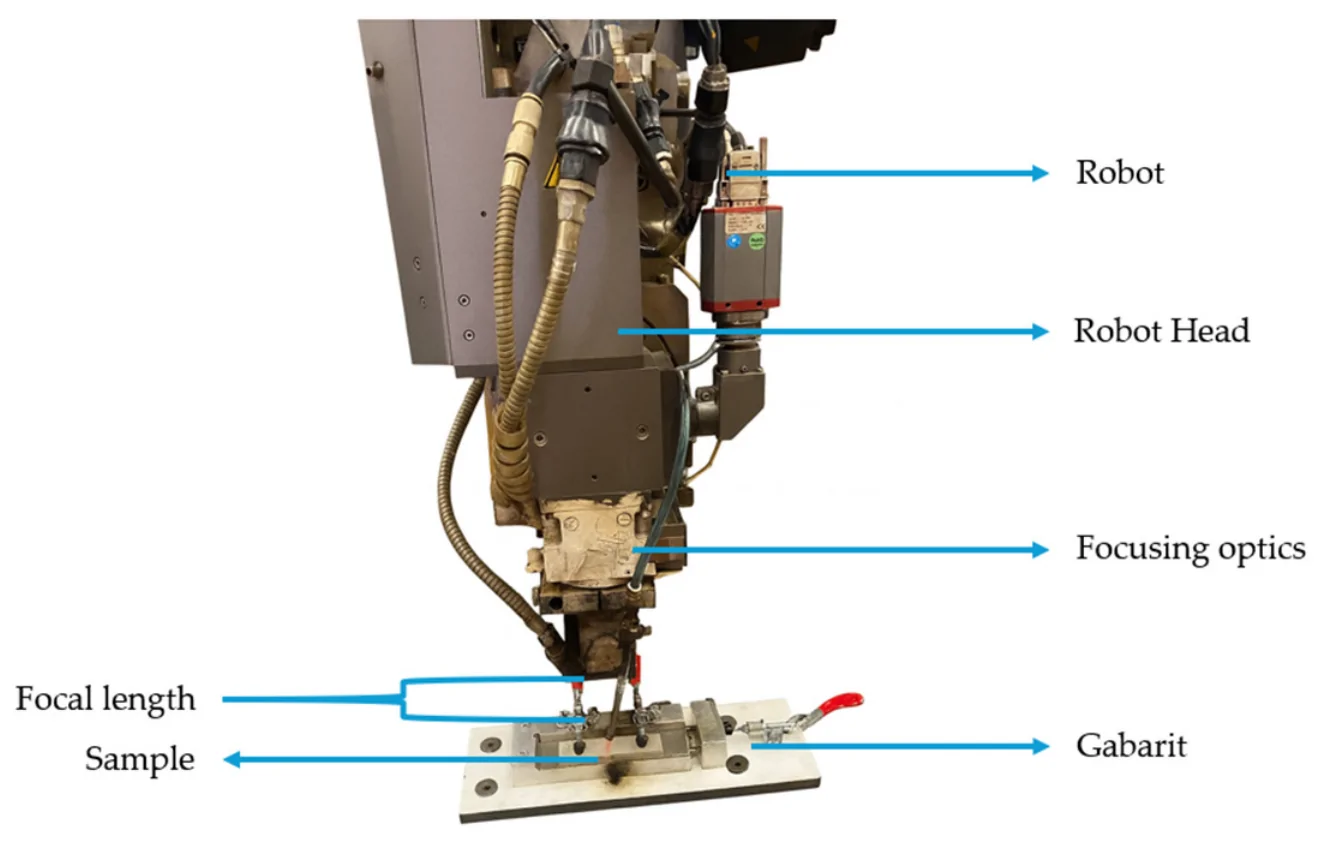
Laser welding robot making multiple passes on PP and PET, Guimarães et al. [11].

The experiments were conducted using the same TRUMPF TruDisk 6602 Nd: YAG disk laser system (TRUMPF, Schramberg, Germany) and a KUKA robotic unit equipped with a BEO D70 optical head (TRUMPF, Schramberg, Germany), as shown in Figure 2.

**Figure 3:**
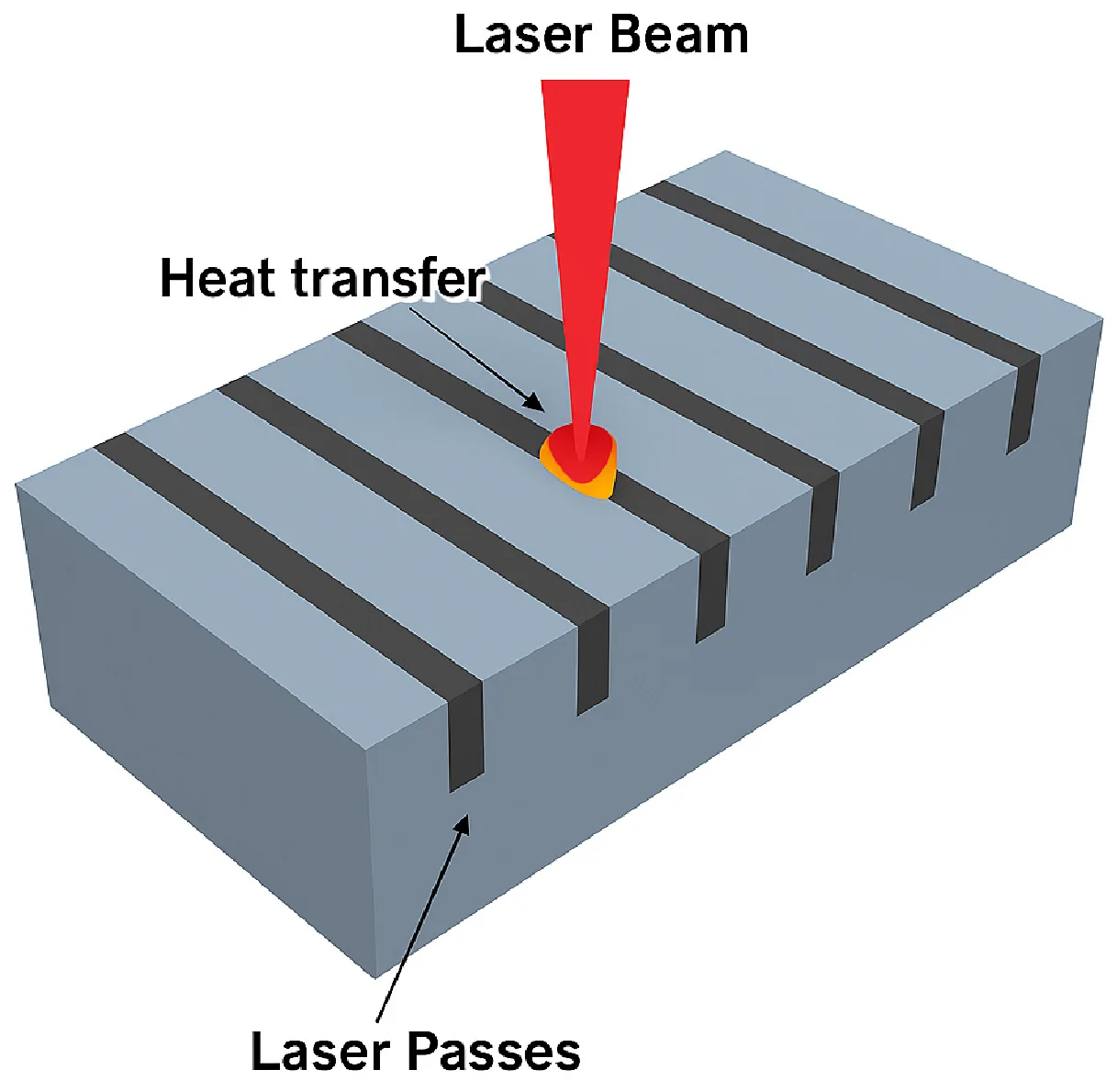
Experimental setup for laser passes on PP and PET sample.

Figure 3 illustrates the experimental configuration adopted for the laser processing of the polymer plates, showing the laser beam directed onto the sample surface and the resulting heat transfer through the material thickness. Multiple parallel laser passes were performed across the plate surface to ensure uniform exposure and to replicate identical processing conditions for both polymers, simulating controlled, repeated energy input during laser treatment.

The main operating parameters of the laser welding robot are summarized in Table 2. This approach replicated the methodology applied by Guimarães et al. [11] for PP, enabling a consistent comparison of the thermal and microstructural responses of PET and PP under identical laser parameters. All optical parameters, including the collimation distance (150 mm), focal distance (10 mm), focused beam diameter (0.4 mm), and focal position (0 mm), were kept constant throughout all experiments to ensure experimental comparability.

### 2.3. Hardness Testing

The hardness analysis followed the same experimental procedure previously described by Guimarães et al. [11]. Measurements were performed along the entire thickness of the specimen, across the bead depth, using a Vickers microhardness tester (HMV-2000, Shimadzu Corporation, Tokyo, Japan), in accordance with the ASTM E384 standard [57]. A constant load of 0.200 kgf (1.961 N) was applied for 15 s using a pyramidal diamond indenter, producing the characteristic indentation marks.

The Vickers microhardness values were measured along with the through-thickness direction of each sample, starting from the upper surface. For every position, three independent measurements (HV_1_, HV_2_, HV_3_) were taken to account for local variations, and their arithmetic mean (HVavg) and standard deviation (HVstd) were calculated. To enable direct comparison between samples, the average and standard deviation values were normalized with respect to the maximum hardness recorded in each test series, resulting in the parameters HV(avg, norm) and HV(std, norm). This normalization ensured consistent comparative analysis between samples by minimizing the influence of experimental variability.

### 2.4. Optical Microscopy

Image acquisition and measurement of the laser-affected region were performed using a high-definition magnification system coupled with a digital camera (model LW5507, Science Experiment Textbook, Nagykáta, Hungary). The images were captured under identical magnification and illumination conditions to ensure consistency. Measurements of the laser extent of the HAZ were carried out directly on the acquired images using calibrated image analysis software.

### 2.5. XRD

The crystalline structure of the PET and PP samples was analyzed by XRD using a Rigaku DMAX III/C diffractometer (Rigaku Holdings Corporation, Tokyo, Japan). The diffraction patterns were acquired in θ–2θ scan mode using Cu Kα radiation (λ = 1.54 Å), at an operating voltage of 40 kV and a current of 30 mA. The scans were collected over a 2θ range from 5° to 50°, with a step size of 0.05°.

The relative crystallinity of the polymers was estimated using the peak deconvolution/area integration approach, where the crystalline contribution was separated from the amorphous halo and the crystallinity index was calculated as Equation (1):

(1) $$X_{c}\;(\%)=\;\frac{A_{C}}{A_{C}+\;A_{a}}\;\times \;100$$

where *A_C_* and *A_a_* correspond to the integrated crystalline and amorphous areas, respectively. The XRD profiles were fitted by peak deconvolution using Gaussian components representing the crystalline reflections and the amorphous halo. The reported R^2^ values were used solely as goodness-of-fit indicators for the XRD peak-deconvolution procedure. Because these values were not intended for statistical inference, confidence intervals were not calculated. The reliability of the fittings was verified through visual inspection of residuals and consistency between independent peak assignments.

### 2.6. FTIR

The samples were analyzed by FTIR in attenuated total reflection (ATR) mode to complement the characterization of the materials. The FTIR spectrum of each material provides a unique profile that can be used for identification and comparison of different materials, like a “fingerprint”. To perform this test, 64 scans were selected, with a resolution of 4 cm^−1^ in the FTIR-ATR spectrometer Thermo Scientific Nicolet^TM^ iS^TM^10 (Thermo Fisher Scientific™, Waltham, MA, USA).

### 2.7. DSC

DSC analyses were performed using a NETZSCH DSC 204 calorimeter (NETZSCH-Gerätebau GmbH Spain Branch Office, Barcelona, Spain). Samples were heated from room temperature to 300 °C under a controlled atmosphere at a constant heating rate. The heating rate was set at 10 °C min^−1^, as this condition represents a suitable compromise between thermal resolution and test duration. The DSC thermograms were recorded as heat flow (mW mg^−1^) as a function of temperature, allowing the identification of thermal transitions and melting behavior. The melting temperature (Tm) was determined from the maximum of the endothermic melting peak. Comparisons between untreated and laser-treated samples were used to evaluate the influence of laser processing on the thermal behavior and crystalline structure of the polymers.

## 3. Results and Discussion

### 3.1. Hardness Analysis

Unlike previous studies that focus on individual polymers, the present work enables a direct comparison between PP and PET under identical processing conditions, allowing the isolation of material-dependent effects on hardness evolution and microstructural response.

As described in the methodology, each average hardness value corresponds to the mean of three independent measurements (HV_1_, HV_2_, HV_3_), subsequently normalized to the maximum value recorded in each test series. This approach ensures a consistent and dimensionless comparison (0–1 scale) between samples, minimizing local variability and enabling a reliable evaluation of hardness evolution through the material thickness.

The experimental results in Table 3 and Table 4 show the normalized Vickers microhardness values measured along the thickness of the PET samples for different scanning speeds (5–50 mm/s) and laser powers (200 W and 300 W). These results illustrate the variation in hardness through the material thickness and how laser power and scanning speed affect the thermal response and microstructural behavior of the polymer.

**Figure 4:**
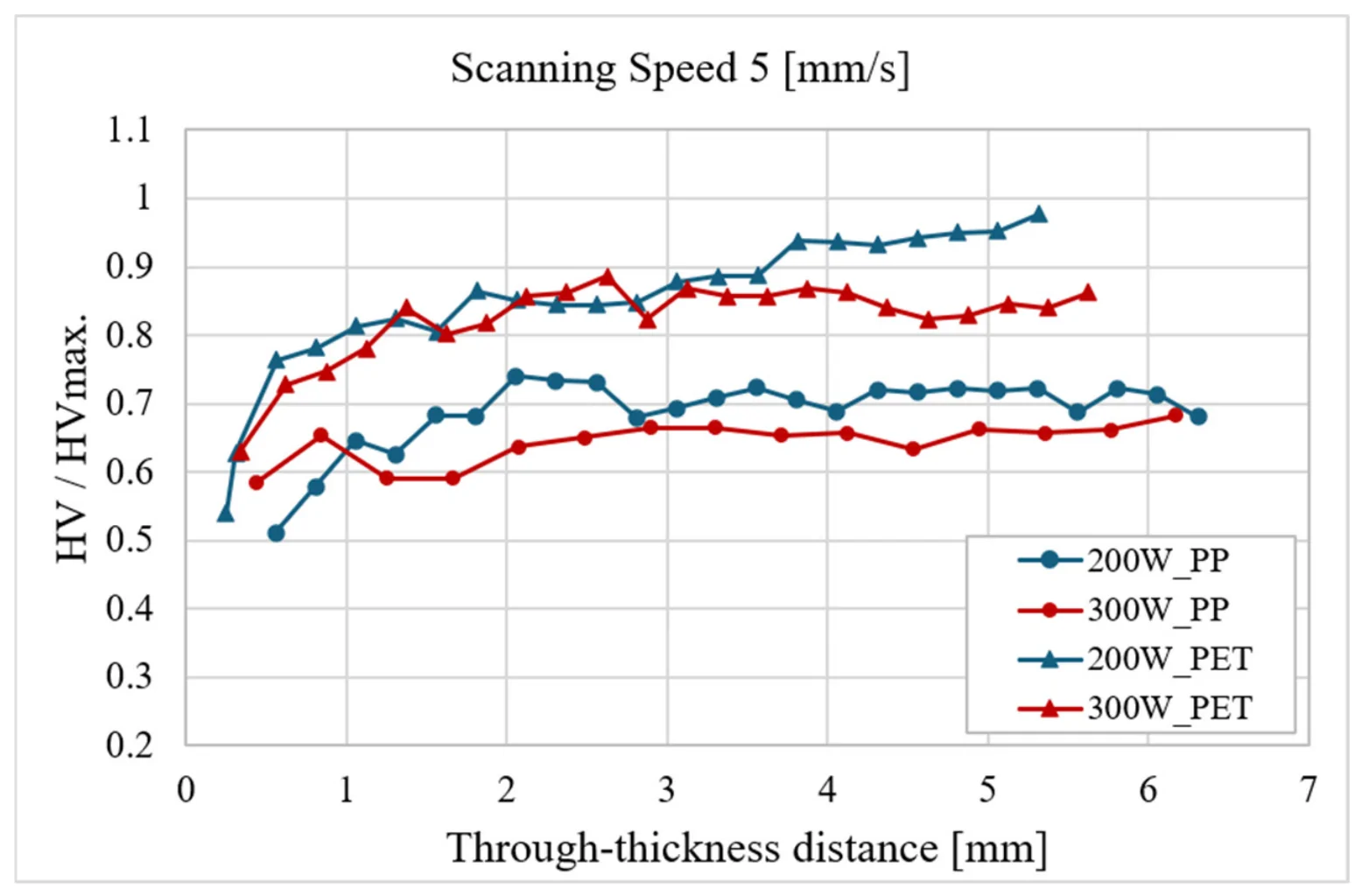
Comparison of hardness evolution through-thickness of PP and PET samples at a scanning speed of 5 mm/s. Average hardness profiles for 200 W and 300 W laser power across the sample thickness.

**Figure 5:**
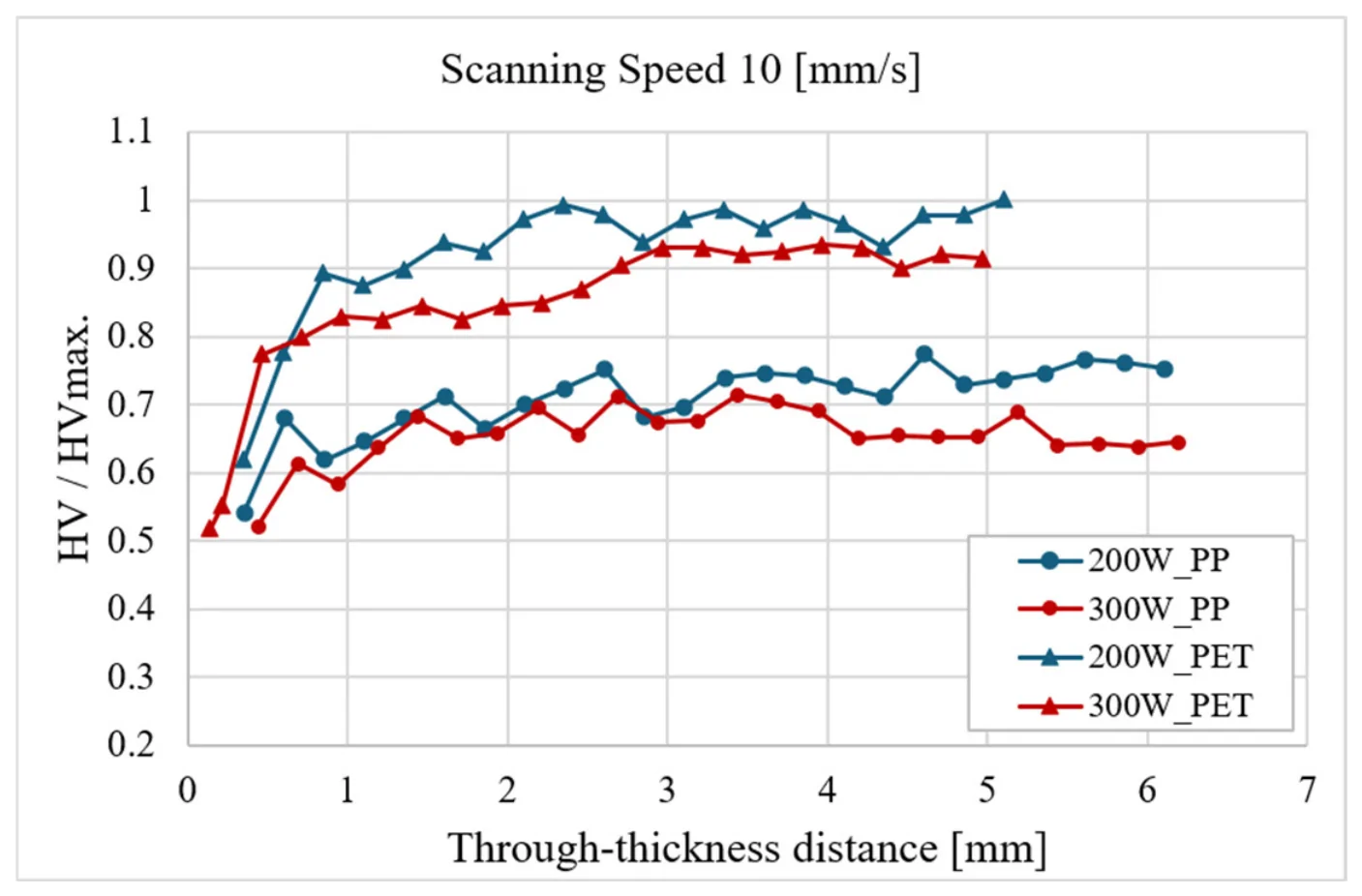
Comparison of hardness evolution through-thickness of PP and PET samples at a scanning speed of 10 mm/s. Average hardness profiles for 200 W and 300 W laser power across the sample thickness.

**Figure 6:**
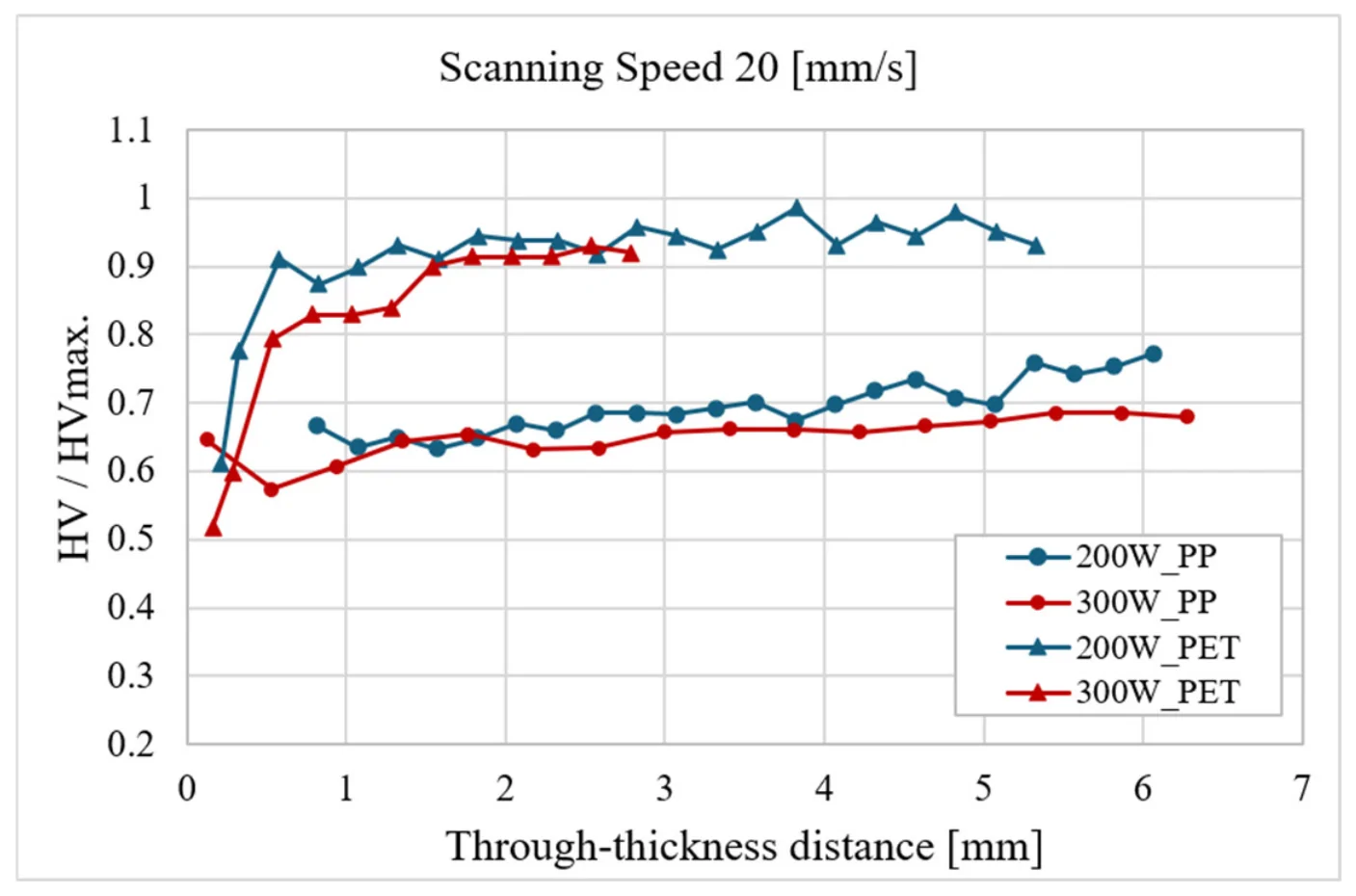
Comparison of hardness evolution through-thickness of PP and PET samples at a scanning speed of 20 mm/s. Average hardness profiles for 200 W and 300 W laser power across the sample thickness.

**Figure 7:**
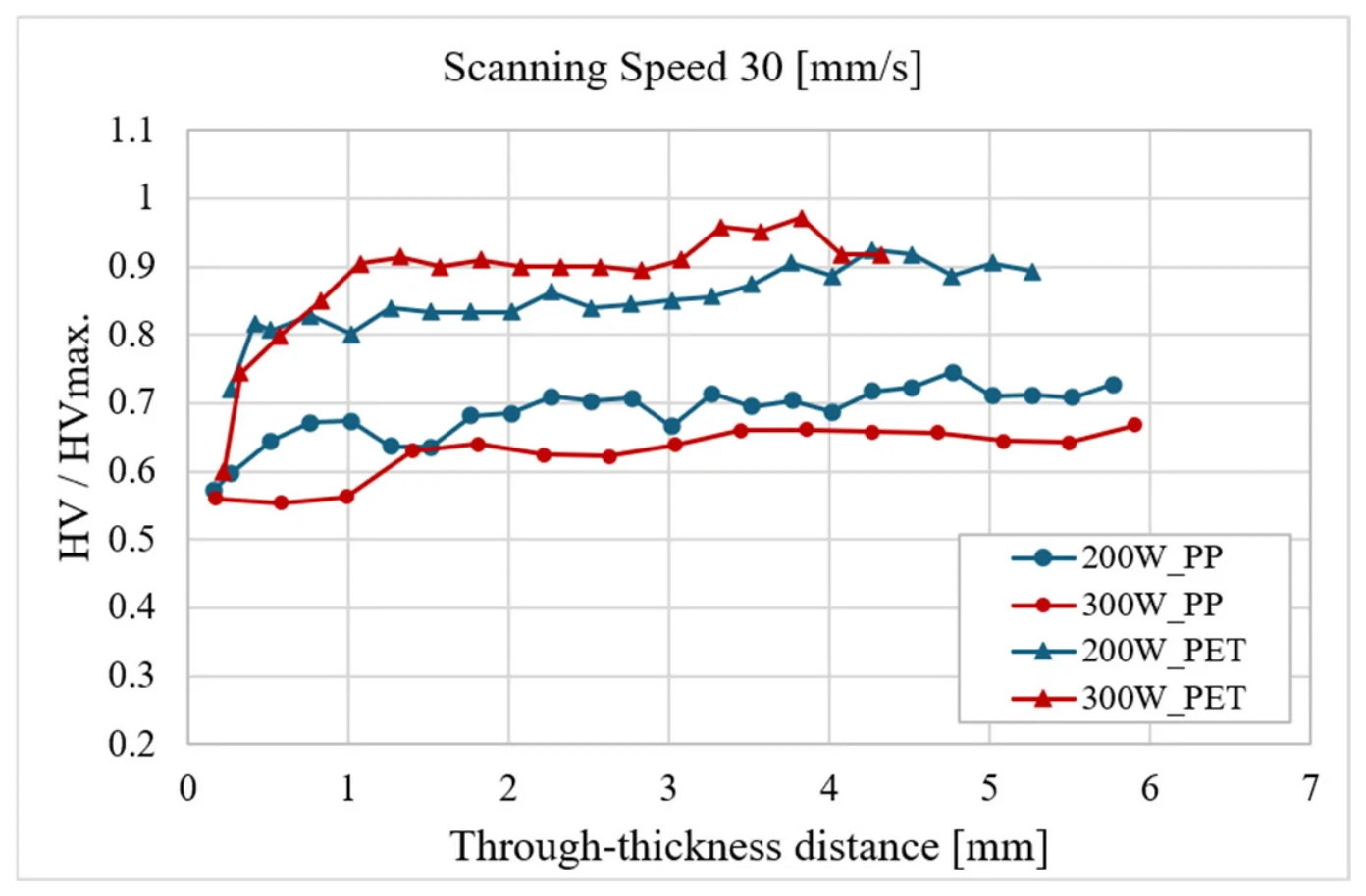
Comparison of hardness evolution through-thickness of PP and PET samples at a scanning speed of 30 mm/s. Average hardness profiles for 200 W and 300 W laser power across the sample thickness.

**Figure 8:**
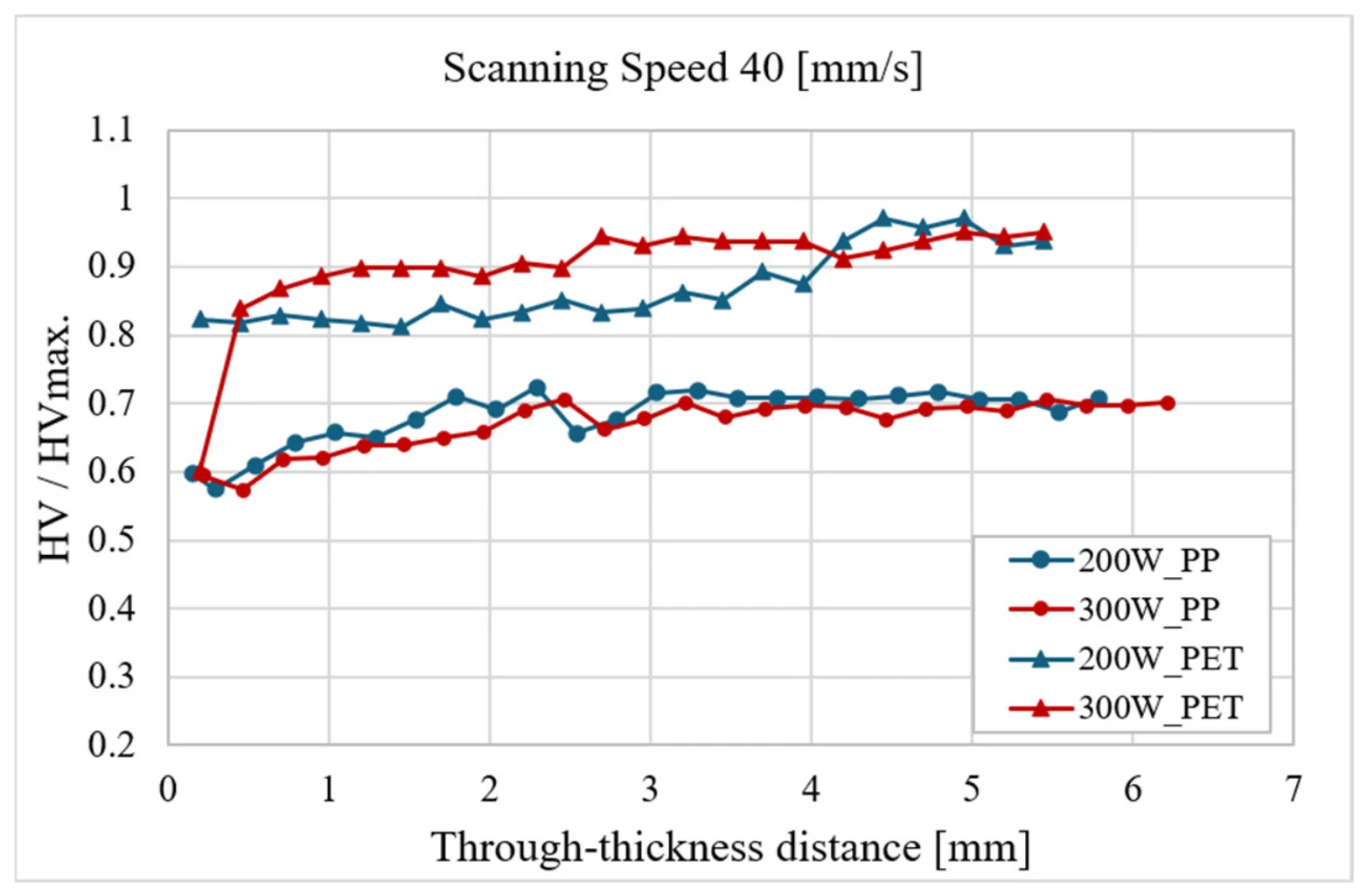
Comparison of hardness evolution through-thickness of PP and PET samples at a scanning speed of 40 mm/s. Average hardness profiles for 200 W and 300 W laser power across the sample thickness.

**Figure 9:**
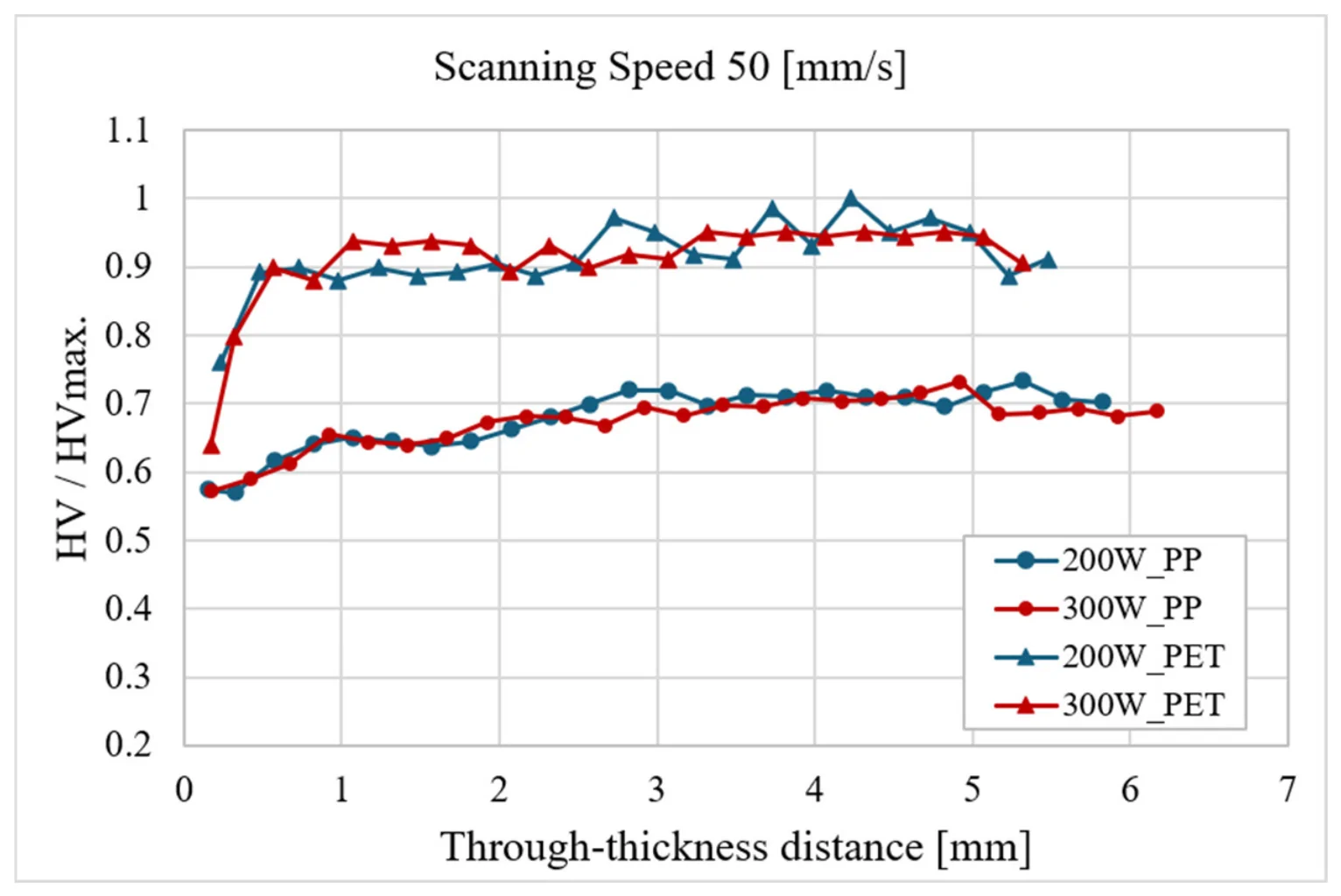
Comparison of hardness evolution through-thickness of PP and PET samples at a scanning speed of 50 mm/s. Average hardness profiles for 200 W and 300 W laser power across the sample thickness.

The comparative assessment between PP and PET reveals distinct through-thickness hardness evolution under identical laser processing conditions, as illustrated in Figure 4, Figure 5, Figure 6, Figure 7, Figure 8 and Figure 9.

Figure 4, Figure 5, Figure 6, Figure 7, Figure 8 and Figure 9 present the through-thickness hardness evolution of PP and PET as a function of scanning speed (5–50 mm/s) and laser power (200 and 300 W).

At the lowest scanning speed (5 mm/s), corresponding to the highest heat input, PP exhibited a pronounced increase in surface hardness followed by stabilization toward the mid-thickness. Increasing the laser power to 300 W led to a reduction and flattening of the hardness profile, indicating surface softening, chain scission, and partial amorphization. In contrast, PET maintained significantly higher and more uniform hardness values across the entire thickness, demonstrating superior resistance to thermal degradation and more consistent recrystallization during cooling. This behavior is attributed to the higher melting (~255 °C) and glass transition (~75 °C) temperatures of PET compared to PP (~165 °C), as well as its lower thermal expansion.

With increasing scanning speed (10 mm/s), both polymers exhibited more homogeneous hardness distributions due to reduced laser–material interaction time. Nevertheless, PP remained sensitive to higher laser power, displaying reduced hardness associated with localized softening. PET, on the other hand, showed minimal variation with depth or power, maintaining high hardness levels throughout the thickness.

At intermediate scanning speeds (20–30 mm/s), further reductions in thermal input led to narrower HAZs and improved hardness uniformity. PP exhibited relatively stable profiles, although a decrease in hardness at 300 W persisted, indicating residual sensitivity to thermal effects. PET consistently demonstrated higher hardness values with negligible dependence on laser power, confirming its greater thermal stability.

At higher scanning speeds (40–50 mm/s), the reduced interaction time minimized heat accumulation, resulting in the most uniform hardness distributions for both materials. Under these conditions, PP approached a more stable thermal regime with reduced gradients; however, it remained slightly affected by laser power. PET exhibited nearly overlapping hardness profiles for both power levels, highlighting its low sensitivity to processing parameters and enhanced structural stability.

Overall, increasing scanning speed improves hardness uniformity in both polymers by limiting excessive heat input. However, PET consistently exhibits higher hardness, smaller gradients, and weaker dependence on laser power, maintaining its semicrystalline integrity under varying processing conditions. This behavior may be associated with differences in optical absorption, thermal conductivity, and crystallization behavior between PET and PP. In contrast, PP remains more susceptible to surface softening and thermal degradation, achieving improved stability primarily through reduced energy input rather than intrinsic thermal resistance.

These differences can be better understood by considering the molecular structure of each polymer. PET contains rigid aromatic rings and polar ester groups, which strengthen intermolecular interactions and enhance its thermal stability, leading to a more stable response under laser heating. In contrast, PP is made up of a fully aliphatic and nonpolar backbone with greater chain mobility, which makes it more susceptible to thermal softening, chain scission, and oxidative degradation when exposed to laser irradiation.

### 3.2. Optical Microscope Measurements

The dimensions of the laser-affected zone were determined from images acquired using a stereomicroscope equipped with a digital camera. The images were captured under identical magnification and illumination conditions to ensure consistency. Measurements of the laser extent of the HAZ were carried out directly on the acquired images using calibrated image analysis software.

**Figure 10:**
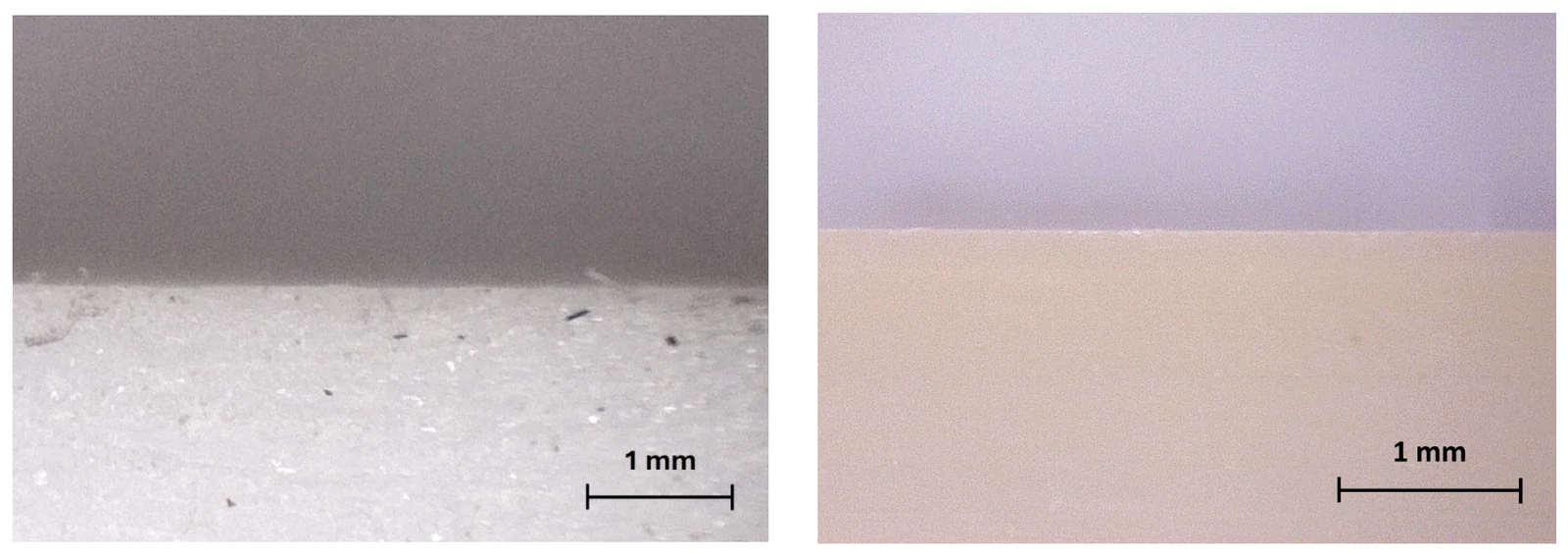
Optical microscopy of virgin PET (**left**) and PP (**right**) samples.

**Figure 11:**
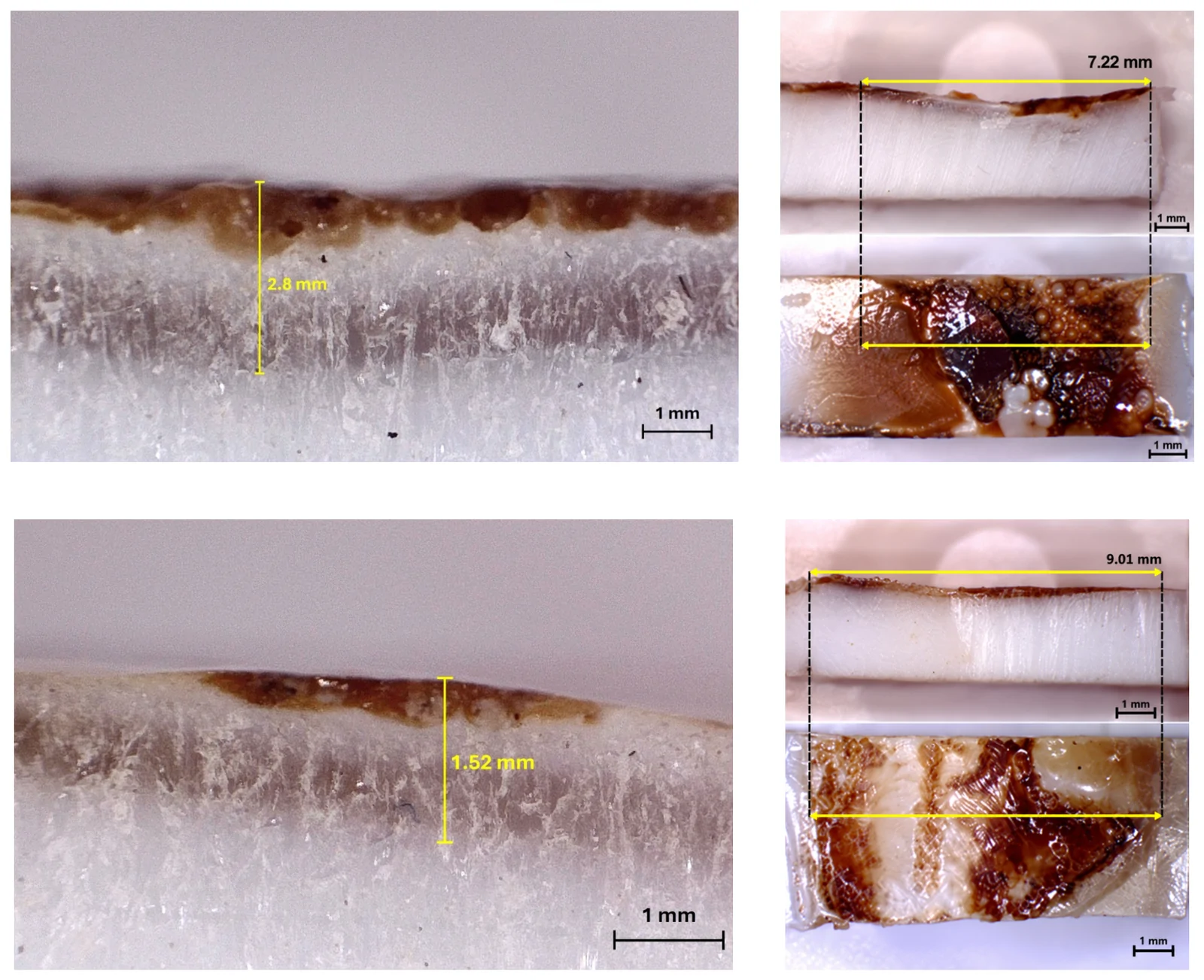
Optical microscopy of the HAZ of PET samples processed at 200 W (**top**) and 300 W (**below**) at 5 mm/s.

**Figure 12:**
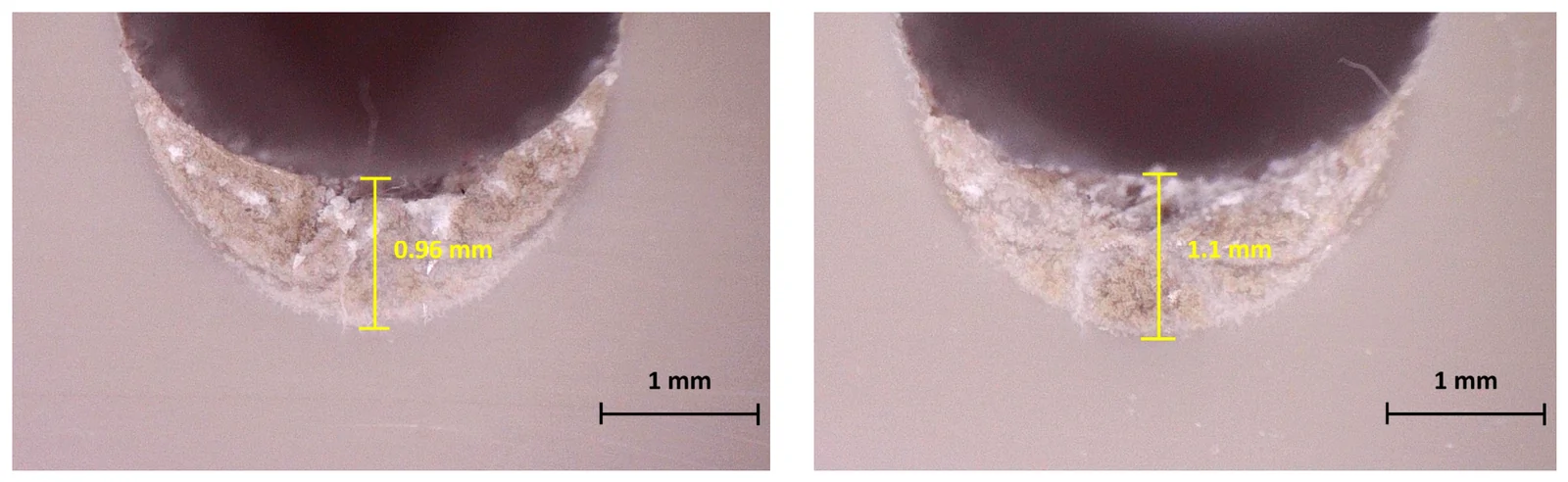
Optical microscopy of the HAZ of PP samples processed at 200 W (**left**) and 300 W (**right**) at 5 mm/s.

The micrographs presented in Figure 10 correspond to the virgin PET and PP samples, while Figure 11 and Figure 12 show the corresponding HAZs after laser processing at the lowest scanning speed (5 mm/s) using powers of 200 W and 300 W, respectively. These conditions were selected to isolate the effect of laser power under maximum heat input.

A clear distinction in laser–material interaction is observed between the two polymers. In PET, both power levels primarily lead to surface burning and bubble formation, with no development of a pronounced molten pool. This indicates that energy absorption is largely confined to the near-surface region. In contrast, PP exhibits the formation of a deep molten pool, reflecting more efficient bulk heat absorption and enhanced material flow.

The geometry of the HAZ in PET further supports this interpretation. At 200 W, the HAZ is deeper (2.8 mm) but narrower (7.22 mm), whereas at 300 W it becomes significantly wider (9.01 mm) and shallower (1.52 mm). This behavior suggests a transition from penetration-dominated to surface-dominated energy dissipation with increasing power. The higher reflectance of PET limits energy coupling into the bulk at elevated power levels, promoting lateral heat spreading and surface degradation rather than deeper melting.

By contrast, PP shows a monotonic increase in HAZ thickness with power (from 0.96 mm at 200 W to 1.1 mm at 300 W), indicating more effective volumetric energy absorption and heat conduction. This promotes bulk melting and deeper thermal penetration, consistent with the observed molten pool formation.

These microstructural observations are directly reflected in the hardness profiles reported in Figure 4, Figure 5, Figure 6, Figure 7, Figure 8 and Figure 9. At low scanning speed (5 mm/s), where heat input is highest, PP shows reduced and flattened hardness profiles at higher power, consistent with extensive melting, chain scission, and thermal degradation within the bulk. Conversely, PET maintains higher and more stable hardness values across the thickness, despite visible surface degradation. This confirms that the thermal effects in PET remain largely superficial and do not significantly compromise subsurface mechanical integrity.

As scanning speed increases (10–50 mm/s), the reduction in interaction time limits heat accumulation in both materials, leading to more uniform hardness profiles. However, the underlying mechanisms remain distinct. In PP, improved hardness uniformity results primarily from reduced thermal damage (i.e., less severe melting and degradation), whereas in PET it reflects inherently stable thermal behavior, with minimal sensitivity to power and consistent recrystallization across the thickness.

These trends are consistent with broader findings in laser processing of semicrystalline polymers. Materials with lower melting temperatures and higher thermal expansion coefficients, such as PP, typically exhibit greater susceptibility to bulk melting, thermal degradation, and hardness reduction under high energy input. In contrast, polymers with higher thermal stability, such as PET, tend to undergo surface-limited modification, preserving bulk mechanical properties while exhibiting localized surface damage. Additionally, increased reflectance and lower optical absorption at certain wavelengths have been reported to promote lateral heat diffusion and reduce penetration depth, further reinforcing surface-dominated behavior. Beyond the laser power window, the spatial distribution of the deposited laser power is itself a design variable. Recent advances in beam engineering have demonstrated that optical field shaping can confine power deposition to sub-diffraction-limit scale, offering a route to decouple penetration depth from spot size in future laser processing of polymers [58].

### 3.3. XRD Analysis

The degree of crystallinity of the virgin PET and PP samples was determined by XRD peak deconvolution and area integration, yielding values of 43.3% and 59.3%, respectively as can be observed in Table 5.

Moreover, the values presented in Table 1 correspond to typical crystallinity ranges reported by the supplier for the bulk commercial materials, whereas the values reported in Table 5 were experimentally determined by XRD peak deconvolution and area integration for the virgin samples analyzed in this study. Consequently, the crystallinity values reported in this study should be considered experimental estimates obtained by XRD peak deconvolution and area integration and interpreted in the context of the materials and analysis procedure adopted. Nevertheless, the obtained values remain within the ranges commonly reported for semicrystalline PET and PP materials processed under conventional industrial conditions. PET is generally described as a semicrystalline polymer with a degree of crystallinity strongly influenced by its thermal history and processing conditions, typically ranging from approximately 30 to 50%, whereas PP commonly exhibits crystallinity values between 50 and 70% [59,60]. These observations support the validity of the measured values while highlighting their use as indicators of relative structural changes resulting from laser treatment.

**Figure 13:**
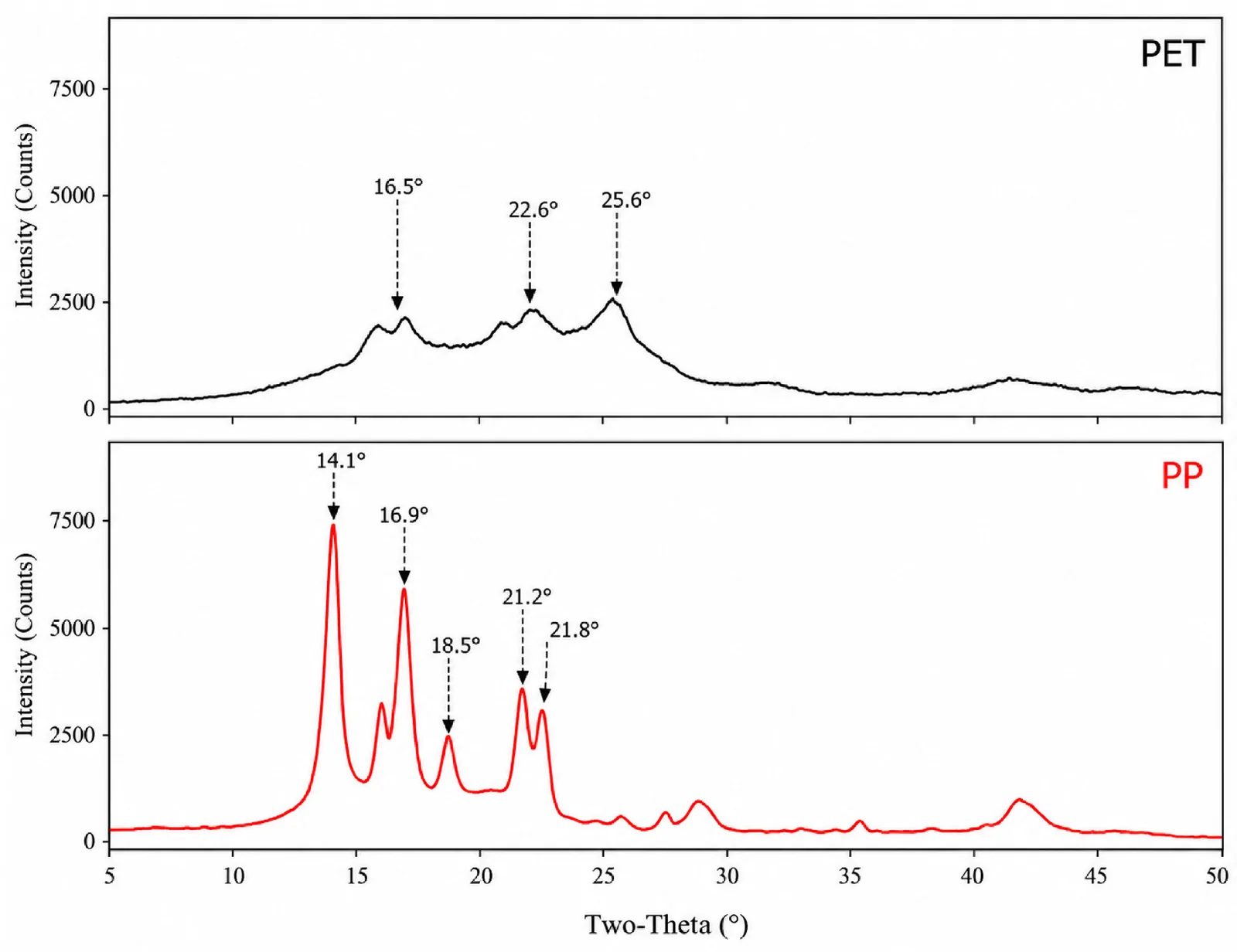
XRD patterns of virgin PET (**top**) and PP (**bottom**), with the main diffraction peaks indicated.

The crystallinity values presented in Table 5 show that both virgin PP and PET exhibit a significant crystalline fraction, with PP displaying a higher crystallinity (59.31%) than PET (43.30%). This difference reflects the distinct molecular structures and crystallization behaviors of the two polymers. The calculated values fall within the ranges commonly reported for commercial semicrystalline PP and PET materials [59,60]. In addition, the high R^2^ values obtained from the peak deconvolution procedure indicate that the crystalline reflections and amorphous contributions were adequately resolved, supporting the reliability of the calculated crystallinity values. These baseline structural characteristics are essential for interpreting the changes observed after laser processing. The XRD patterns of virgin PET and PP samples, with their main diffraction peaks identified, are shown in Figure 13.

Following the establishment of the reference crystal structure of virgin PP and PET samples, the next step involves evaluating the effect of laser processing on their microstructural evolution. The XRD results presented below show the changes in crystallinity induced by different processing conditions, specifically laser powers of 200 W and 300 W, combined with scan speeds ranging from 5 mm/s, as illustrated in Table 6. These diffractograms enable the evaluation of the microstructural evolution of PET after laser processing, while the virgin PP and PET samples provide a baseline comparison between both materials. The comparative analysis highlights the influence of laser parameters on peak intensity, peak broadening, and overall crystallinity, providing a deeper understanding of the structural mechanisms and mechanical behaviors discussed in previous sections.

For the laser-treated samples, distinct crystallization behaviors were observed. The PET_200W sample exhibited a crystallinity of 35.69%, lower than that measured for virgin PET (43.30%). This reduction suggests that laser processing promoted partial disruption of the ordered crystalline domains and increased the relative amorphous fraction. Localized heating followed by rapid cooling may hinder crystal growth and induce structural disorder in semicrystalline polymers, resulting in lower crystallinity values [59].

In contrast, PET_300W exhibited a crystallinity of 52.15%, corresponding to an increase relative to the untreated material. This behavior suggests that the thermal energy introduced during laser processing was sufficient to promote molecular rearrangement and recrystallization. PET crystallization is highly sensitive to thermal treatments, and previous studies have reported that appropriate thermal exposure can enhance chain mobility and facilitate crystal growth, leading to increased crystallinity [59].

The opposite trends observed for PET_200W and PET_300W indicate that the processing conditions may place the material in different crystallization regimes. Under conditions dominated by rapid cooling, the formation of amorphous regions is favored, as observed for PET_200W. Conversely, when sufficient chain mobility is achieved before solidification, secondary crystallization and crystal growth may occur, leading to higher crystallinity, as observed for PET_300W. Similar behavior has been reported for semicrystalline PET subjected to thermal processing [59,60].

The goodness-of-fit values obtained from the deconvolution procedure (R^2^ = 0.943 for PET_200W and R^2^ = 0.906 for PET_300W) indicate the fit achieved when separating the crystalline reflections from the amorphous contribution.

Overall, at fixed exposure time (slow scan), a clear hierarchy is observed:

*PET_200W low power (~35%) < Virgin PET (~43%) ≪ PET_300W high power (~52%).*

In general, at fixed exposure time (slow scan), the results reveal distinct crystallization responses depending on the laser power applied. PET_200W exhibited a lower crystallinity (~35%) than the virgin material (~43%), indicating that laser-induced melting followed by rapid cooling promoted the formation of a larger amorphous fraction. In contrast, PET_300W reached the highest crystallinity (~52%), suggesting that the higher thermal input provided sufficient molecular mobility to favor recrystallization and crystal growth during cooling.

These observations indicate that laser processing may drive PET into different crystallization regimes. Under lower power conditions, thermal history appears to favor crystalline disruption and amorphization, whereas higher power promotes molecular rearrangement and the development of a more ordered structure. As crystallinity increases, stiffness, hardness, and dimensional stability are generally expected to improve, although often at the expense of toughness and ductility. Consequently, laser power emerges as a critical parameter governing the balance between structural ordering and amorphization in laser-treated PET, with direct implications for the long-term performance of joined or modified components.

In summary, the results demonstrate that the crystalline structure of PET can be tailored through laser processing. Depending on the thermal input, laser irradiation may either reduce or enhance crystallinity relative to virgin material, providing a practical means of controlling microstructural development and, consequently, the final material properties.

**Figure 14:**
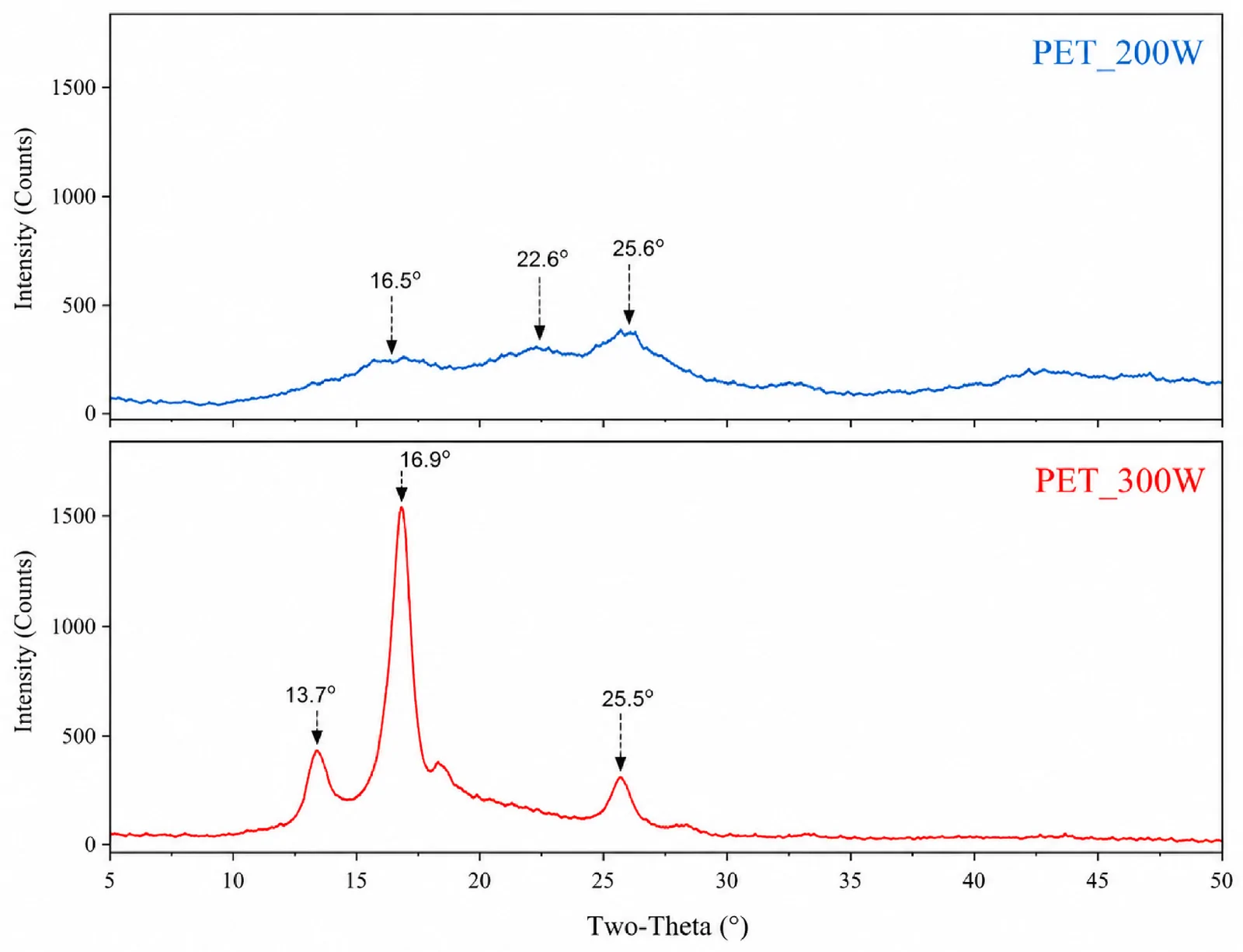
XRD patterns of PET samples processed at 200 W (**top**) and 300 W (**bottom**) at a scanning speed of 5 mm/s, with the main diffraction peaks indicated.

Figure 14 presents the deconvoluted diffraction patterns used for crystallinity determination, showing the separation between crystalline reflections and the amorphous halo. Variations in the relative contributions of these components, compared with the virgin material, reflect the influence of laser processing on melting, recrystallization, and structural rearrangement within the polymer structure.

Overall, the comparative XRD analysis highlights the distinct microstructural response of PP and PET to laser irradiation. PET undergoes localized melting and recrystallization, resulting in either a decrease or an increase in crystallinity depending on the applied laser power (Figure 14). These structural observations are complemented by the FTIR and DSC analysis presented in the following sections, which provide further insight into the chemical modifications induced by laser processing and help establish the relationship between crystalline evolution and polymer degradation.

### 3.4. FTIR Analysis

**Figure 15:**
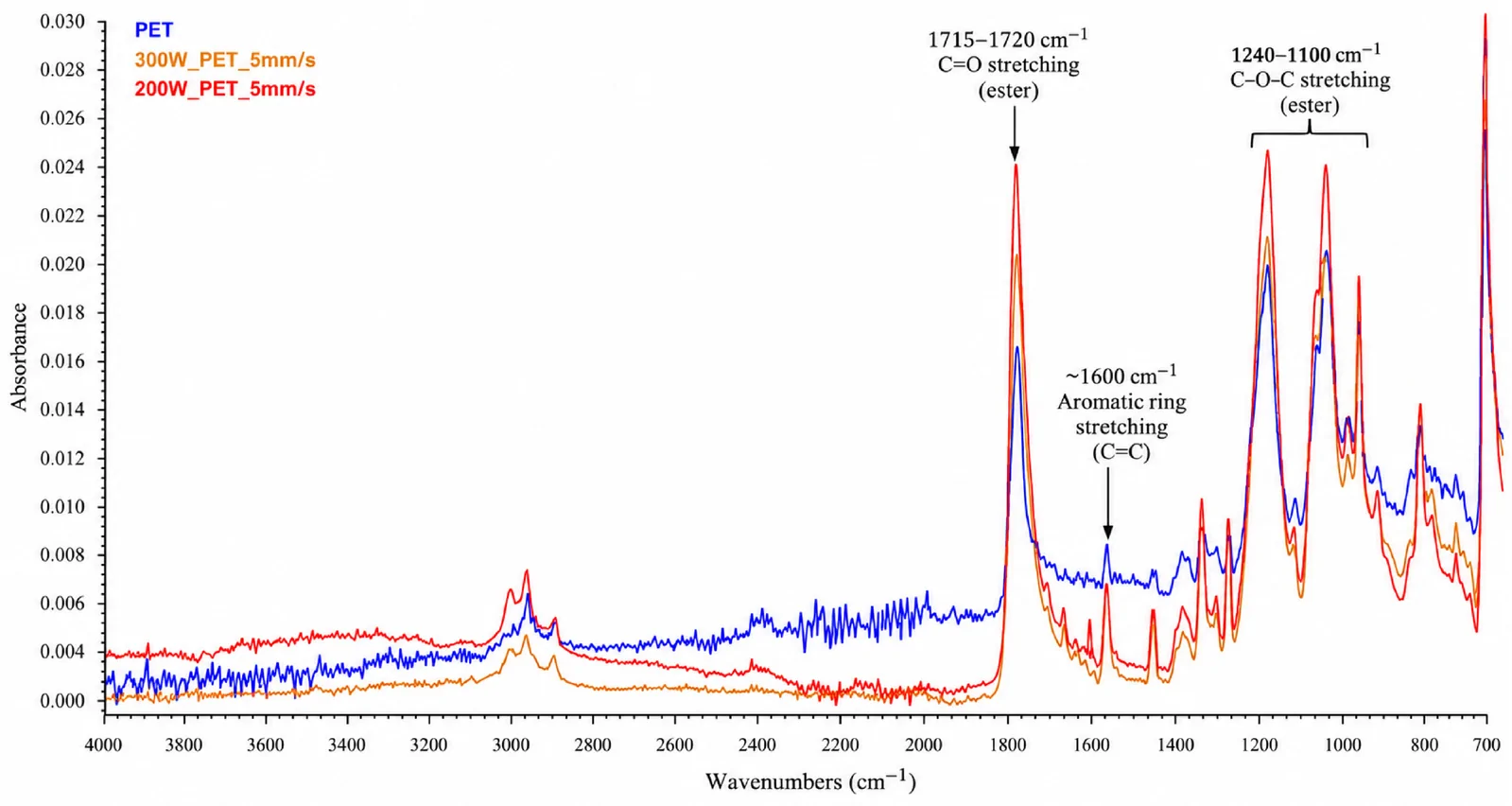
FTIR spectra of PET samples before and after laser processing at 200 W and 300 W at a scanning speed of 5 mm/s, with the main characteristic absorption bands indicated.

The FTIR spectra of PET processed at the lowest scanning speed (5 mm/s) under both laser power levels retain all characteristic vibrational features of the polymer, indicating that the molecular backbone remains chemically intact even under the most thermally demanding conditions (Figure 15). The dominant absorption band at 1715–1720 cm^−1^, corresponding to the stretching vibration of the ester carbonyl (C=O) group, remains unchanged in both position and intensity relative to the virgin material. This band is widely recognized as the most reliable spectroscopic marker for PET integrity and is highly sensitive to ester cleavage or hydrolysis, neither of which is observed here [37,61].

Additional characteristic bands, aromatic ring stretching modes at ~1600 and 1580 cm^−1^, C–O–C asymmetric and symmetric stretching vibrations in the 1240–1100 cm^−1^ region, and out of plane aromatic C–H bending below 900 cm^−1^, also remain well preserved. At 200 W and 5 mm/s, only minor intensity variations are observed in the C–O stretching region, without the appearance of new peaks or band broadening. Similar behavior has been reported in thermally annealed and laser heated PET, where localized heating primarily induces lamellar reorganization and crystallinity enhancement rather than chemical degradation [9,25,45].

When the laser power is increased to 300 W, the spectral profile remains essentially unchanged, with no new absorption bands appearing in the 3200–3600 cm^−1^ region that would indicate hydroxyl formation from hydrolysis or oxidation. The absence of these features confirms that PET does not undergo significant chain scission or oxidative degradation, even under high local energy density. Instead, a slight sharpening of the carbonyl and aromatic bands is detected, which is commonly associated with increased molecular ordering and improved crystalline symmetry [25,29].

These FTIR observations correlate directly with the XRD results obtained for PET, particularly at 5 mm/s. XRD analysis shows an increase in crystallinity for PET_300W, reaching 52.15%, compared with 43.30% for the virgin material, indicating localized melting followed by recrystallization. Importantly, this substantial crystallinity increase is not accompanied by chemical changes in the FTIR spectra, confirming that PET’s response to laser processing is dominated by physical reorganization rather than chemical modification. Similar FTIR–XRD correlations have been reported in PET subjected to laser transmission welding and thermal annealing, where increased peak intensity and sharpening in XRD coincide with preserved FTIR signatures [7,45,48].

Overall, the combined FTIR and XRD evidence demonstrates that PET accommodates high thermal input through controlled melting–recrystallization cycles, resulting in a more ordered semicrystalline structure without compromising chemical integrity. This spectroscopic stability provides a strong basis for the higher hardness retention and microstructural homogeneity observed in PET under identical processing conditions.

In contrast, the FTIR spectra of PP show a higher sensitivity to laser processing at low scanning speed, reflecting the inherently lower thermal and oxidative stability of its aliphatic backbone. It should also be noted that commercial thermoplastics may contain antioxidant and stabilizer packages designed to limit thermo-oxidative degradation during processing and service. Recent studies have demonstrated that antioxidant systems can significantly influence the thermo-oxidative response of both PP and PET by limiting oxidative degradation and radical formation [62,63]. Since the specific additive packages of the commercial grades investigated in the present study were not disclosed by the manufacturer, their potential contribution to the observed thermal and spectroscopic response cannot be independently assessed. The reference PP spectrum is characterized by intense C–H stretching absorptions at 2950–2835 cm^−1^, CH_3_ and CH_2_ bending modes at ~1455 and 1375 cm^−1^, and skeletal vibrations below 1200 cm^−1^, all of which are clearly visible after laser treatment. A very weak shoulder around 1720 cm^−1^ can be discerned in the as-received PP spectrum; however, no distinct carbonyl band is present. This weak feature is attributed to trace carbonyl-containing species commonly found in commercial PP grades as a result of processing, storage, or slight surface oxidation, rather than laser-induced degradation.

At 200 W and 5 mm/s, the characteristic PP bands remain essentially unchanged, indicating that the primary polymer chain structure is largely preserved. However, subtle reductions in the intensity of the CH_3_ deformation band at ~1375 cm^−1^ are observed, together with a slight increase in baseline noise. Such changes have been widely attributed to increased chain mobility, partial melting of the amorphous phase, and early stages of thermo-oxidative sensitivity rather than advanced degradation [27,44,64].

At 300 W and 5 mm/s, more evident spectral changes emerge. Although a very weak shoulder can be discerned around 1720 cm^−1^ in the as-received PP spectrum, no well-defined carbonyl absorption band is present. After processing at 200 W the spectrum remains essentially unchanged, whereas at 300 W a broader absorption develops in the 1700–1800 cm^−1^ region. Although considerably weaker than the characteristic ester carbonyl band of PET, this feature is frequently reported in thermally aged or laser-processed PP and is associated with the initial formation of carbonyl-containing oxidation products, such as ketones and aldehydes [27,30,65].

In addition, a weak broad absorption extending from approximately 3200 to 3600 cm^−1^ becomes discernible after processing at 300 W. This region is commonly attributed to O–H stretching vibrations associated with hydroxyl-containing oxidation products and/or adsorbed moisture. Although of low intensity, its appearance together with the broad carbonyl absorption provides further evidence of the onset of thermo-oxidative processes under the highest laser energy input.

**Figure 16:**
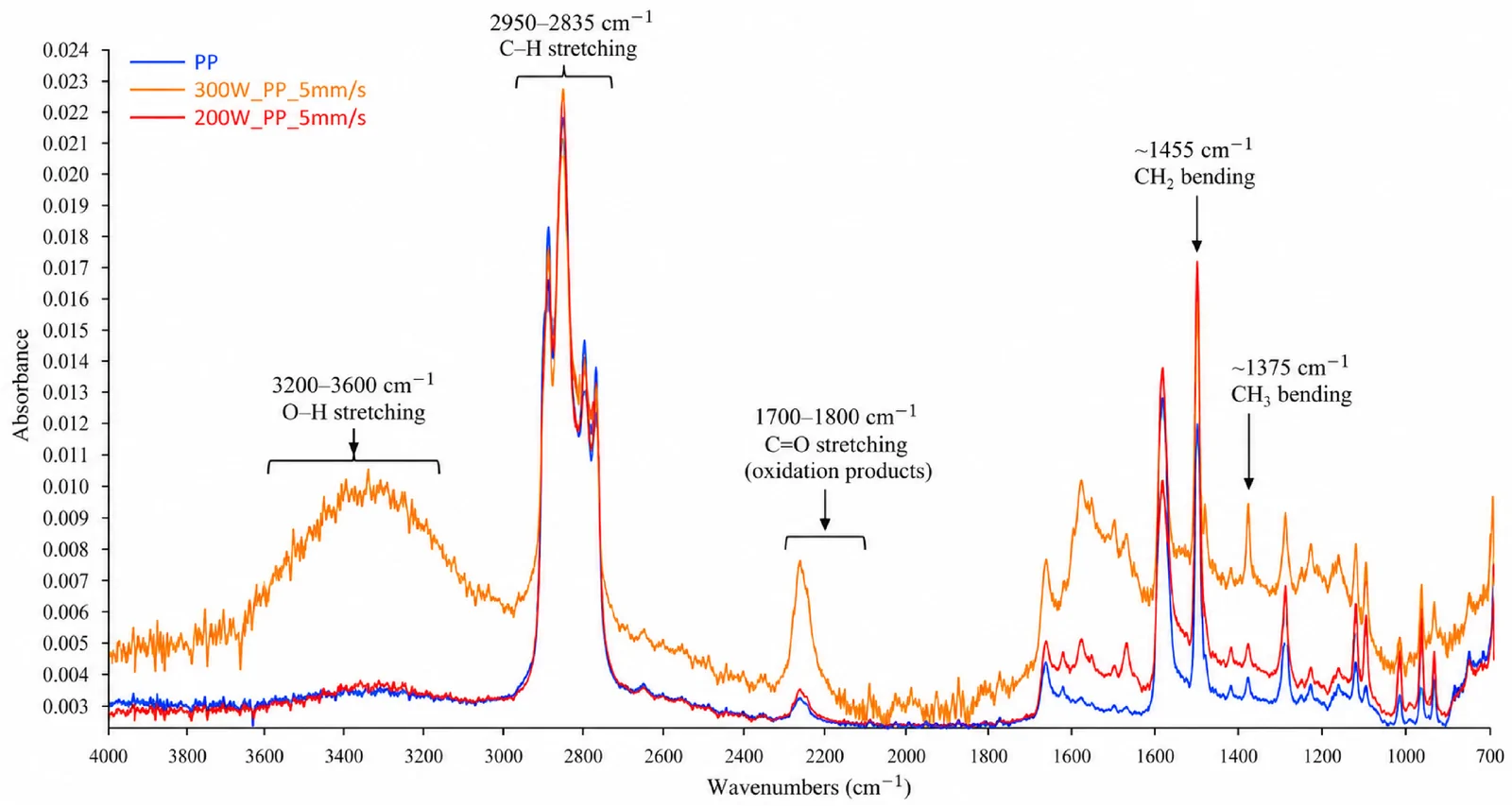
FTIR spectra of PP samples before and after laser processing at 200 W and 300 W at a scanning speed of 5 mm/s, with the main characteristic absorption bands indicated.

Concurrently, small reductions in the intensities of the C–H stretching bands suggest partial disruption of the aliphatic chain environment, consistent with early chain scission or branching reactions under elevated temperature. The onset of chemical signatures associated with oxidation in FTIR coincides with a reduction in hardness and less stable crystallinity values, particularly at 300 W (Figure 16). This coupling between spectroscopic evidence of degradation and structural instability detected by XRD is well documented for PP subjected to high thermal loads, where re-crystallization competes with chain scission and oxidative processes [44,64].

Unlike PET, PP does not exhibit peak sharpening in FTIR that would indicate improved crystalline symmetry. Instead, the spectroscopic evidence points to a delicate balance between recrystallization and degradation, with the latter becoming increasingly relevant at higher laser power under prolonged exposure.

Taken together, FTIR and XRD provide a coherent picture of how the two polymers respond differently to laser processing at low scanning speed. PET shows a consistent alignment between FTIR stability and XRD crystallinity enhancement, confirming that its response is dominated by physical rearrangement of the semicrystalline architecture. PP, in contrast, exhibits early chemical sensitivity in FTIR that may contribute to changes in crystallinity and mechanical performance under higher laser energy input [66]. This combined spectroscopic–structural analysis reinforces the conclusions drawn from microhardness measurements: PET maintains mechanical integrity through recrystallization without chemical damage, whereas PP approaches a degradation threshold where chemical stability becomes a limiting factor. Similar FTIR–XRD correlations have been reported for laser processed semicrystalline polymers and underline the importance of jointly interpreting vibrational and diffraction data when assessing laser–material interactions [45,64].

### 3.5. DSC Analysis

The DSC analysis revealed clear differences between the thermal behavior of the virgin and laser-treated polymeric materials as can be observed in Table 7.

The virgin PP sample exhibited a melting peak centered at approximately 167 °C, which agrees well with the melting temperature range commonly reported for isotactic PP (160–170 °C), the most widely used semicrystalline polypropylene grade in industrial applications [67]. The ΔHm values ranged from approximately 14 to 18 J/g, indicating the presence of a significant crystalline fraction.

After laser treatment, a slight shift in the melting peak toward lower temperatures was observed for PP_200W and PP_300W. Such behavior may indicate minor modifications in crystal perfection and lamellar thickness induced by the localized thermal cycle generated by laser irradiation. The relatively small variation in Tm suggests that the overall crystalline structure of PP remained largely preserved after treatment.

For PET, the virgin material exhibited a melting peak at approximately 246 °C, which is characteristic of semicrystalline PET and falls within the melting temperature range commonly reported for PET in the literature (approximately 245–260 °C) [59,68]. The PET_200W sample exhibited a Tm very similar to that of the untreated material, indicating that laser processing caused limited changes in the crystalline domains. In contrast, PET_300W displayed a slightly higher Tm, reaching approximately 249 °C. This increase may be associated with the formation of more stable crystalline structures or an increase in crystal perfection promoted by laser-induced thermal rearrangement. The estimated ΔHm values for PET varied between 9 and 12 J/g. The DSC and XRD results provide complementary information regarding the structural modifications induced by laser processing. The PET_200W sample showed a lower crystallinity degree than virgin PET, suggesting partial disruption of crystalline regions, whereas PET_300W exhibited increased crystallinity, indicating that the laser treatment promoted chain mobility and recrystallization. The higher Tm observed for PET_300W further supports the hypothesis of improved crystal organization following laser exposure.

For PP, the lack of significant variation in Tm suggests that the laser treatment mainly affected the surface layer and did not substantially alter the bulk crystalline morphology. The DSC results therefore indicate that PET is more sensitive to the thermally induced structural modifications generated by laser processing than PP under the investigated conditions. The higher Tm observed for PET compared with PP reflects the greater chain rigidity and stronger intermolecular interactions associated with the aromatic structure of PET.

The DSC and XRD results provide complementary information regarding the structural evolution of the polymers after laser irradiation. Although PET_300W exhibited a lower melting enthalpy, XRD indicated increased crystallinity. This apparent discrepancy may arise because XRD is primarily sensitive to crystalline ordering, whereas DSC measures the overall melting enthalpy associated with the crystalline fraction present in the analyzed sample. Consequently, differences in crystal perfection, crystal size distribution, or localized ordering may be detected differently by the two techniques.

The combined DSC and XRD analyses suggest that laser irradiation can induce distinct structural responses depending on the polymer type and processing parameters. In PET, laser treatment may either reduce or increase crystallinity depending on the balance between crystal disruption and recrystallization. In contrast, PP maintains a relatively stable crystalline structure, with only minor variations in melting behavior. These findings demonstrate that the thermal history imposed by laser processing plays a critical role in determining the final microstructure and thermal performance of semicrystalline polymers.

## 4. Conclusions

This study provides a systematic comparison of PP and PET under identical laser processing conditions, demonstrating distinct responses to laser-induced thermal input. PP was more sensitive to processing conditions, particularly at low scanning speeds and higher power, exhibiting pronounced through-thickness hardness gradients and surface softening. PET showed more uniform hardness profiles and lower sensitivity to laser power, despite localized surface damage. Increasing the scanning speed reduced thermal exposure and resulted in more uniform hardness distributions in both polymers.

XRD, FTIR, and DSC analyses confirmed that these mechanical responses were accompanied by different structural and chemical changes. PET exhibited changes in crystallinity and thermal behavior consistent with laser-induced structural reorganization, without clear spectroscopic evidence of significant chemical degradation. In contrast, PP showed early signs of thermo-oxidative modification at higher energy input.

Overall, the results demonstrate that laser processing conditions should be optimized according to the specific thermal and structural response of each polymer, with scanning speed playing a particularly important role in controlling thermal input. These findings provide a basis for selecting suitable processing conditions for PP and PET and support further investigation of laser–polymer interactions for welding and surface modification applications.

## Figures and Tables

**Table 1:** Properties of the PP and PET adopted for this work.

Property	Value (PP)	Value (PET)	Unit
Density	920	1380	kg/m^3^
Flammability	HB UL94	HB UL94	-
Typical crystallinity range (supplier data)	30–60	25–55	%
Young’s Modulus	1380	2800	MPa
Yield Strength	30	85	MPa
Tensile Strength	33	75	MPa
Hardness (Shore D)	73	75	Scale D
Coefficient of Thermal Expansion (CTE)	160 × 10^−6^	200 × 10^−6^	K^−1^
Thermal Conductivity	0.22	0.28	W.m^−1^.K^−1^
Continuous Service Temperature (CST)	0–100	−20–115	°C
Melting Temperature	162–167	250–260	°C

**Table 2:** Laser welding robot parameters, Guimarães et al. [11].

Property	Value	Unit
Wave length *λ*	1030 ± 10	nm
Focal Distance	10	mm
Collimation Distance	150	mm
Beam Diameter	0.4	mm
Laser Power	200, 300	W
Speed	5, 10, 20, 30, 40, 50	mm/s
Focal Position (Defocus)	0	mm

**Table 3:** Average hardness and standard deviation across the thickness for different scanning speeds at 200 W laser power, for the PET samples.

50 [mm/s]			40 [mm/s]			30 [mm/s]			20 [mm/s]			10 [mm/s]			5 [mm/s]		
Dist. [mm]	HV avg	HV std	Dist. [mm]	HV avg	HV std	Dist. [mm]	HV avg	HV std	Dist. [mm]	HV avg	HV std	Dist. [mm]	HV avg	HV std	Dist. [mm]	HV avg	HV std
0.231	0.761	0.167	0.199	0.823	0.047	0.266	0.719	0.134	0.216	0.612	0.302	0.349	0.619	0.309	0.250	0.539	0.311
0.481	0.893	0.036	0.449	0.817	0.052	0.415	0.817	0.035	0.327	0.776	0.138	0.599	0.776	0.152	0.314	0.628	0.222
0.731	0.899	0.029	0.699	0.828	0.041	0.514	0.807	0.046	0.577	0.911	0.003	0.849	0.893	0.035	0.564	0.764	0.086
0.981	0.880	0.048	0.949	0.823	0.047	0.764	0.828	0.025	0.827	0.874	0.040	1.099	0.874	0.053	0.814	0.782	0.068
1.231	0.899	0.029	1.199	0.817	0.052	1.014	0.801	0.051	1.077	0.899	0.015	1.349	0.899	0.029	1.064	0.813	0.036
1.481	0.886	0.041	1.449	0.812	0.058	1.264	0.839	0.013	1.327	0.931	0.016	1.599	0.937	0.009	1.314	0.824	0.025
1.731	0.893	0.036	1.699	0.845	0.024	1.514	0.834	0.019	1.577	0.911	0.003	1.849	0.924	0.004	1.564	0.805	0.044
1.981	0.905	0.023	1.949	0.823	0.047	1.764	0.834	0.019	1.827	0.944	0.030	2.099	0.972	0.044	1.814	0.865	0.015
2.231	0.886	0.041	2.199	0.834	0.036	2.014	0.834	0.019	2.077	0.937	0.023	2.349	0.993	0.065	2.064	0.851	0.001
2.481	0.905	0.023	2.449	0.851	0.019	2.264	0.862	0.010	2.327	0.937	0.023	2.599	0.979	0.051	2.314	0.845	0.005
2.731	0.972	0.044	2.699	0.834	0.036	2.514	0.839	0.013	2.577	0.918	0.003	2.849	0.937	0.009	2.564	0.845	0.005
2.981	0.951	0.023	2.949	0.839	0.030	2.764	0.845	0.007	2.827	0.958	0.044	3.099	0.972	0.044	2.814	0.848	0.002
3.231	0.918	0.010	3.199	0.862	0.007	3.014	0.851	0.002	3.077	0.944	0.030	3.349	0.986	0.058	3.064	0.878	0.029
3.481	0.911	0.017	3.449	0.851	0.019	3.264	0.857	0.004	3.327	0.924	0.010	3.599	0.958	0.030	3.314	0.886	0.036
3.731	0.986	0.058	3.699	0.893	0.023	3.514	0.874	0.022	3.577	0.951	0.037	3.849	0.986	0.058	3.564	0.887	0.038
3.981	0.931	0.003	3.949	0.874	0.005	3.764	0.905	0.052	3.827	0.986	0.072	4.099	0.965	0.037	3.814	0.938	0.088
4.231	1.000	0.072	4.199	0.937	0.068	4.014	0.886	0.034	4.077	0.931	0.016	4.349	0.931	0.003	4.064	0.937	0.087
4.481	0.951	0.023	4.449	0.972	0.102	4.264	0.924	0.071	4.327	0.965	0.051	4.599	0.979	0.051	4.314	0.933	0.083
4.731	0.972	0.044	4.699	0.958	0.088	4.514	0.918	0.065	4.577	0.944	0.030	4.849	0.979	0.051	4.564	0.942	0.092
4.981	0.951	0.023	4.949	0.972	0.102	4.764	0.886	0.034	4.827	0.979	0.064	5.099	1.000	0.072	4.814	0.950	0.100
5.231	0.886	0.041	5.199	0.931	0.061	5.014	0.905	0.052	5.077	0.951	0.037				5.064	0.953	0.103
5.481	0.911	0.017	5.449	0.937	0.068	5.264	0.893	0.040	5.327	0.931	0.016				5.314	0.977	0.128

**Table 4:** Average hardness and standard deviation across the thickness for different scanning speeds at 300 W laser power, for the PET samples.

50 [mm/s]			40 [mm/s]			30 [mm/s]			20 [mm/s]			10 [mm/s]			5 [mm/s]		
Dist. [mm]	HV avg	HV std	Dist. [mm]	HV avg	HV std	Dist. [mm]	HV avg	HV std	Dist. [mm]	HV avg	HV std	Dist. [mm]	HV avg	HV std	Dist. [mm]	HV avg	HV std
0.170	0.638	0.270	0.199	0.602	0.300	0.222	0.598	0.282	0.162	0.518	0.307	0.141	0.518	0.327	0.339	0.630	0.194
0.319	0.799	0.110	0.449	0.839	0.062	0.324	0.744	0.136	0.288	0.598	0.227	0.215	0.553	0.292	0.625	0.728	0.096
0.569	0.899	0.010	0.699	0.868	0.033	0.574	0.799	0.081	0.538	0.794	0.031	0.465	0.774	0.071	0.875	0.747	0.077
0.819	0.880	0.028	0.949	0.886	0.015	0.824	0.849	0.031	0.788	0.829	0.004	0.715	0.799	0.045	1.125	0.781	0.043
1.069	0.937	0.029	1.199	0.899	0.002	1.074	0.905	0.025	1.038	0.829	0.004	0.965	0.829	0.015	1.375	0.839	0.016
1.319	0.931	0.022	1.449	0.899	0.002	1.324	0.915	0.035	1.288	0.839	0.014	1.215	0.824	0.020	1.625	0.801	0.022
1.569	0.937	0.029	1.699	0.899	0.002	1.574	0.899	0.020	1.538	0.899	0.075	1.465	0.844	0.000	1.875	0.817	0.006
1.819	0.931	0.022	1.949	0.886	0.015	1.824	0.910	0.030	1.788	0.915	0.090	1.715	0.824	0.020	2.125	0.857	0.033
2.069	0.893	0.016	2.199	0.905	0.004	2.074	0.899	0.020	2.038	0.915	0.090	1.965	0.844	0.000	2.375	0.862	0.039
2.319	0.931	0.022	2.449	0.899	0.002	2.324	0.899	0.020	2.288	0.915	0.090	2.215	0.849	0.005	2.625	0.886	0.063
2.569	0.899	0.010	2.699	0.944	0.043	2.574	0.899	0.020	2.538	0.930	0.105	2.465	0.869	0.025	2.875	0.823	0.001
2.819	0.918	0.009	2.949	0.931	0.029	2.824	0.894	0.015	2.788	0.920	0.095	2.715	0.905	0.060	3.125	0.868	0.045
3.069	0.911	0.003	3.199	0.944	0.043	3.074	0.910	0.030				2.965	0.930	0.085	3.375	0.857	0.033
3.319	0.951	0.042	3.449	0.937	0.036	3.324	0.958	0.078				3.215	0.930	0.085	3.625	0.857	0.033
3.569	0.944	0.036	3.699	0.937	0.036	3.574	0.951	0.071				3.465	0.920	0.075	3.875	0.868	0.045
3.819	0.951	0.042	3.949	0.937	0.036	3.824	0.972	0.092				3.715	0.925	0.080	4.125	0.862	0.039
4.069	0.944	0.036	4.199	0.911	0.010	4.074	0.918	0.038				3.965	0.935	0.090	4.375	0.839	0.016
4.319	0.951	0.042	4.449	0.924	0.023	4.324	0.918	0.038				4.215	0.930	0.085	4.625	0.823	0.001
4.569	0.944	0.036	4.699	0.937	0.036							4.465	0.899	0.055	4.875	0.828	0.004
4.819	0.951	0.042	4.949	0.951	0.049							4.715	0.920	0.075	5.125	0.845	0.021
5.069	0.944	0.036	5.199	0.944	0.043							4.965	0.915	0.070	5.375	0.839	0.016
5.319	0.905	0.004	5.449	0.951	0.049										5.625	0.862	0.039

**Table 5:** Crystallinity results for virgin PP and PET samples using XRD peak deconvolution and area integration.

Material	Crystalline Area (%)	Amorphous Area (%)	Crystallinity (%)	R^2^
PP	59.31	40.69	59.31	0.898
PET	43.30	56.70	43.30	0.985

**Table 6:** Crystallinity results for PET samples processed at 200 W and 300 W with a scanning speed of 5 mm/s, using XRD peak deconvolution and area integration.

Material	Crystalline Area (%)	Amorphous Area (%)	Crystallinity (%)	R^2^
PET_200W	35.69	64.31	35.69	0.943
PET_300W	52.15	47.85	52.15	0.906

**Table 7:** Influence of laser processing on the Peak Melting Temperature (Tm) and Melting Enthalpy (ΔHm) of PP and PET samples.

Sample	Melting Temperature, Tm (°C)	Melting Enthalpy, ΔHm (J/g)
Virgin PP	167	18
PP_200W	165	15
PP_300W	164	14
Virgin PET	245	12
PET_200W	245	10
PET_300W	249	9

## Data Availability

The original contributions presented in this study are included in the article. Further inquiries can be directed to the corresponding author.
